# Venous thromboembolism risk in adults with hereditary thrombophilia: a systematic review and meta-analysis

**DOI:** 10.1007/s00277-024-05926-2

**Published:** 2024-08-21

**Authors:** Anne B. Alnor, Charlotte Gils, Pernille J. Vinholt

**Affiliations:** 1 Deptartment of Clinical Biochemistry, Odense University Hospital, Odense, Denmark; 2 Department of Clinical Research, University of Southern Denmark, Odense, Denmark

**Keywords:** Venous thrombosis, Thrombophilia, Protein C, Antithrombins, Protein S

## Abstract

**Abstract:**

This systematic review and meta-analysis assesses venous thromboembolism (VTE) risk in adults with hereditary thrombophilia, including Factor V Leiden (FVL) mutation, prothrombin G20210A (FII) mutation, compound heterozygosity, protein C (PC), protein S (PS), and antithrombin (AT) deficiency. Eligibility criteria included studies suitable for quantitative synthesis with extractable information on VTE risk in adults (> 15 years). There were no restrictions on VTE type, location, or occurrence. Two authors reviewed all studies and extracted data from 107 publications, encompassing 107,130 individuals (21,560 experiencing VTE). We used a random effects model and calculated odds ratios (ORs) with 95% confidence intervals (CIs). The highest risk was associated with homozygous FVL (OR 5.58, 95% CI 4.61–6.74), homozygous FII (OR 5.16, 95% CI 3.12–8.52), and compound heterozygosity (OR 4.64, 95% CI 2.25–9.58). In contrast, VTE risk was lowest for FVL heterozygosity (OR 2.97, 95% CI 2.41–3.67) and FII heterozygosity (OR 2.21, 95% CI 1.70–2.87), whereas PC (OR 3.23, 95% CI 2.05–5.08), PS (OR 3.01, 95% CI 2.26–4.02), and AT deficiency (OR 4.01, 95% CI 2.50–6.44) demonstrated an intermediate VTE risk. These results highlight an increased risk of venous thromboembolism in adults with hereditary thrombophilia. However, the risk for patients with PC, PS, and AT deficiency appears to be lower than previously stated, likely due to varying thrombogeneity of the underlying genetic mutations. Further research addressing this aspect of VTE risk in hereditary thrombophilia is imperative to improve patient management.

**Trial registration:**

PROSPERO registration number CRD42022376757.

**Supplementary Information:**

The online version contains supplementary material available at 10.1007/s00277-024-05926-2.

## Introduction

Venous thromboembolism (VTE) is a prevalent and preventable cause of death, significantly affecting patients’ quality of life [1, 2]. Intrinsic and environmental risk factors may lead to VTE through several different mechanisms that are frequently overlapping [3]. Hereditary thrombophilia is a common risk factor in VTE, often associated with unprovoked VTE in younger patients [4]. Existing literature on VTE-risk in hereditary thrombophilia is extensive with contrasting results, warranting systematic reviews to extrapolate accurate risk estimates. Previously published systematic reviews are either not up to date [5], or examine specific subgroups such as pregnant women [6], children [7], or patients with particular conditions [8]. Our objective is to provide a contemporary assessment of VTE risk in adults with: Factor V Leiden mutation (hetero- and homozygous), prothrombin G20210A mutation (hetero- and homozygous), Factor V Leiden and prothrombin compound heterozygosity, and natural anticoagulant deficiency (protein S, protein C, and antithrombin deficiency).

## Methods

### Data sources and search strategy

We searched PubMed, Embase (Ovid), and Web of Science on the 17th of November 2022 for articles on hereditary thrombophilia. No date or language restrictions were applied. A repeat search on the 15th of November 2023, targeted relevant articles published in the interim year. The supplementary material provides the search strategy and hit count. We reviewed the references of included studies to identify any articles overlooked by our search.

### Eligibility criteria

Cohort and case-control studies in English, German, Danish, Swedish, or Norwegian, providing extractable information on VTE risk in adults (> 15 years) with hereditary thrombophilia (HT), were eligible for inclusion. Only studies suitable for quantitative synthesis were included, with no restrictions on VTE type, location, or occurrence (i.e. primary or recurrent). Studies were eligible regardless of the method used for diagnosing VTE, but adherence to objective diagnostic criteria was an element of the quality assessment of studies (see supplementary). Genetic confirmation was required for Factor V Leiden (FVL) and prothrombin G20210A (FII), while protein C (PC), protein S (PS), and antithrombin (AT) deficiency did not require genetic diagnostics, aligning with international recommendations [9–11]. Therefore, PC, PS, or AT deficiency was defined as protein levels below the diagnostic cut-offs. We excluded: single-family studies, studies on genetically secluded populations, studies on the interaction between hormonal treatment and HT, studies exclusively on pregnancy/ pregnancy–related outcomes, studies set in intensive care, studies dealing with specific comorbidities or surgical treatment of patients with HT, and studies where the type of HT could not be discerned (e.g. not disclosed if patients were heterozygous or homozygous for FVL). The full list of eligibility criteria is available in the supplementary information.

### Selection process

We used the Covidence tool (www.covidence.org) throughout the selection process, including identification and removal of duplicate publications. Two authors (CG and AA) independently conducted screening and full-text review of all publications. Disagreements were solved by discussion, and if consensus could not be reached, the third reviewer (PJV) was consulted. Publications were included or excluded based on the predefined eligibility criteria. Authors of publications were not contacted to retrieve or specify information.

### Data collection process and data items

We extracted data in duplicate using a standardised form (see supplementary). Discrepancies and disagreements were resolved by involving a third reviewer. Automation tools were not used for data extraction. We grouped family studies as cohort studies for both meta-analysis and quality assessment. In both case-control and in cohort studies, we recorded (1) the number of VTE patients and their thrombophilia (as applicable), and (2) the number of non-VTE patients and their thrombophilia (as applicable). We also noted the occurrence of VTE occurrence (primary, recurrent, both, or undisclosed) and the location of VTE when available. Superficial venous thromboses (SVT) were included as VTE events. For cohort studies investigating recurrent thrombosis, individuals with recurrent events were considered VTE patients and those without recurrence served as controls.

Across all studies, we documented sex, age, comorbidities, anticoagulant or antithrombotic treatment, and the use of oral contraceptives or hormone replacement therapy due to their associations with VTE risk. We recorded all comorbidities mentioned in the studies. When the information on these factors was not provided, we noted “not stated”. If a study excluded patients based on comorbidity or medication, we noted “not applicable”.

### Risk of bias assessment

We applied the Newcastle-Ottawa Scale (NOS) for quality assessment in both case-control and cohort studies [12]. The complete NOS used is available in the supplementary information. All studies were evaluated for population selection (max. four stars) and comparability of study individuals (max. two stars). Case-control studies were further assessed for exposure (max. two stars), while cohort studies were evaluated for outcome (max. three stars). Overall, studies were assessed based on (1) method of VTE diagnosis, (2) representativeness of VTE and non-VTE individuals, (3) control for additional major VTE risks other than thrombophilia, and (4) ascertainment of thrombophilia diagnosis. The maximum overall score was eight for case-control studies and nine for cohort studies, with a minimum score of zero for all studies. We categorised studies as low risk of bias (overall score 7–9), intermediate risk of bias (overall score 4–6), and high risk of bias (overall score 0–3).

### Statistical analysis

Computations and graphics were performed using Meta-Essentials (Erasmus Research Institute of Management) [13]. We employed a random effects model and calculated odds ratios (ORs) with 95% confidence intervals (CIs) as the effect size measure, using Mantel-Haenzel as the weighting method. Forest plots display individual study ORs along with the overall pooled estimate. For heterogeneity assessment, we used I^2^ and tau squared (τ^2^). I^2^ quantifies the proportion of total variability in effect estimates attributable to heterogeneity rather than chance. τ^2^ was calculated using the DerSimonian and Laird method, representing the estimated between-study variance in the random-effects model. Significant heterogeneity was predefined as > 50% for I^2^ and > 0.50 for τ^2^.In studies with significant heterogeneity, we conducted subgroup analyses based on study design (case-control vs. cohort). Subgroup analyses for studies with low risk of bias (NOS score ≥ 7) and VTE occurrence (primary vs. recurrent) were performed irrespective of heterogeneity. Sensitivity analysis was not conducted. Studies where VTE type was unspecified or indiscernible were grouped for subgroup analysis. We also reported 95% prediction intervals (PIs) of the combined effect size. Prediction interval describe the effect size of a new study selected randomly from the same population as the meta-analysis population. For publication bias analysis, we (1) created funnel plots illustrating the relationship between study effect sizes and the combined effect size, and (2) conducted Egger’s test to quantitatively assess funnel plot asymmetry. An Egger’s test intercept significantly different from 0 with a p-value of < 0.05 suggests publication bias. Funnel plot asymmetry was not assessed for subgroup analyses with fewer than 10 included studies.

## Results

### Summary of selection process

**Fig. 1:**
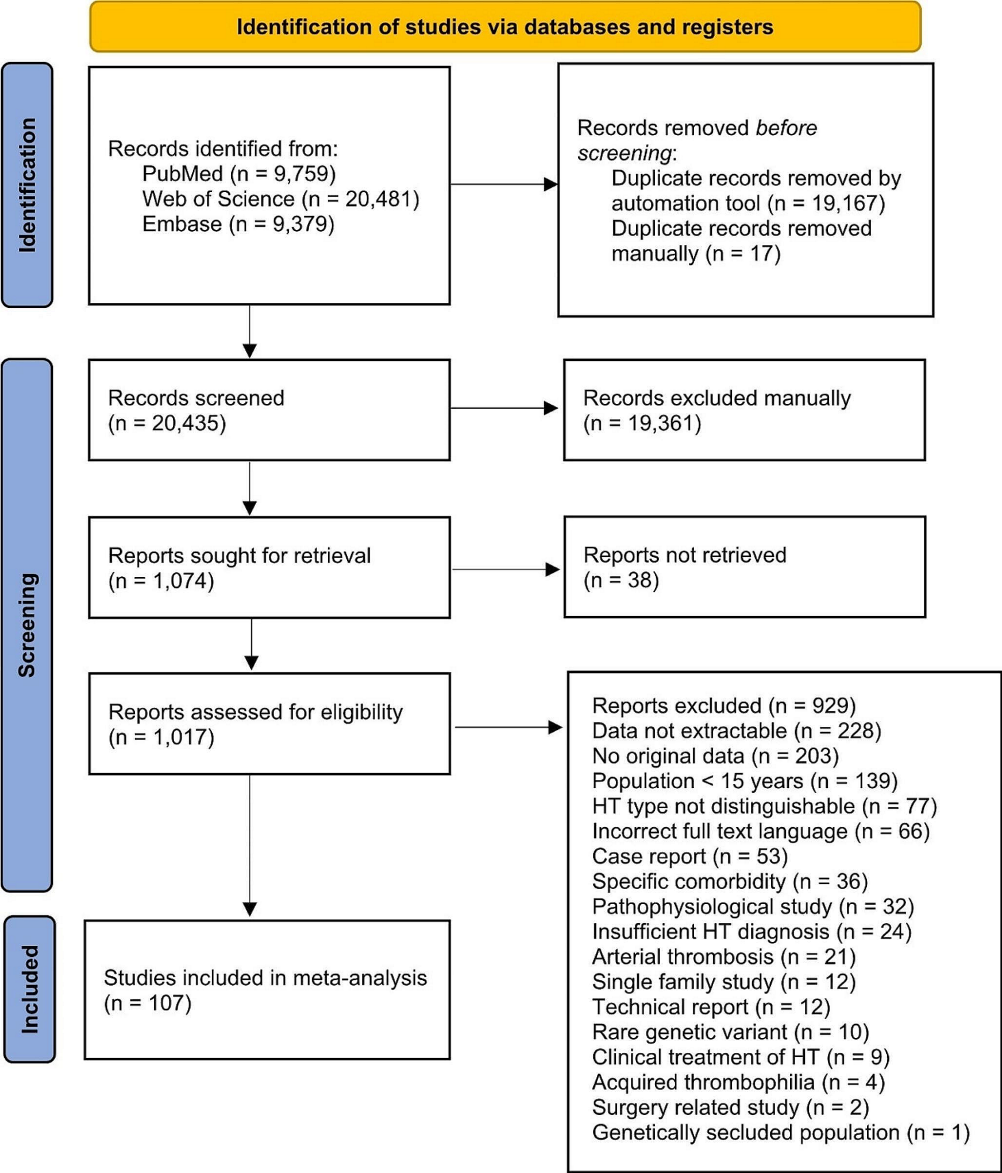
PRISMA Flow-diagram showing number of publications identified, included, and excluded, with reasons for exclusion

Figure 1 shows the selection process. Our search yielded 39,619 publications, of which 19,184 were duplicates. Following title and abstract screening, we assessed 1,017 publications for eligibility, of which 107 studies (76 case-control and 31 cohort studies) were included in the meta-analysis. Most publications reported on several thrombophilias. An overview of the details of the studies included for each thrombophilia is available in the supplementary information.

### Study characteristics

The 107 studies were conducted in 38 different countries between 1990 and 2023, with 18.7% published after 2015 (see supplementary Table 1). The median ages for VTE and non-VTE patients were 46.6 years (interquartile range, IQR 40.1–55.7) and 45.5 years (IQR 38.0–54.0), respectively, with 46.3% of VTE patients being male. Most studies (64.5%) included patients consecutively. Primary VTE was reported in 22 studies, recurrent VTE in 18, while the remaining studies had a mix or undisclosed VTE status. The studies evaluated 107,130 individuals, among whom 21,560 experienced a VTE. Hereditary thrombophilia was present in 22.7% of VTE patients. Deep venous thrombosis (DVT) was the most frequent VTE (see supplementary Table 1). The majority of studies (*n* = 79) reported comorbidities in their study population, but information was lacking in most studies regarding anticoagulant use (*n* = 62) and hormonal treatment (*n* = 54) (see supplementary Table 1).

### Risk of bias in studies

The supplementary information provides details on the NOS scores for each study. Among cohort studies (*n* = 31), the median NOS score was 7 (range 3–9), with 19 studies classified as having low risk of bias, 11 as intermediate, and one as high. For case-control studies (*n* = 76), the median NOS score was 5 (range 1–8); ten studies had low risk of bias, 51 had intermediate, and 15 had high risk of bias. Most studies (*n* = 76) scored high in terms of applying adequate methods for VTE and HT diagnosis. Risk of bias was higher concerning population selection, with 44 studies scored low in this category. Moreover, 62 studies either did not control for any major additional VTE risk factor or did not state if such measured were taken, resulting in low scores for comparability.

### Heterozygous and homozygous FVL

Heterozygous FVL was evaluated in 68,939 individuals, of whom 8,687 had the thrombophilia (Table 1). The overall OR for VTE was 2.97 (95% CI 2.41–3.67, 95% PI 0.51–17.49). Risk of primary VTE was 2.74 (1.59–4.75) and 2.16 (1.16–4.03) for recurrent VTE. Heterogeneity was low for primary and recurrent VTE risk, but otherwise high (Table 1). For homozygous FVL, 62,722 patients were evaluated, with 338 being homozygous for FVL. The overall OR for VTE was 5.58 (95% CI 4.61–6.74, 95% PI 4.61–6.74). Risk of primary VTE was 7.38 (95% CI 4.68–11.65) and 3.82 (95% CI 0.87–16.67) for recurrent VTE. Heterogeneity was low and subgroup analyses based on study design were not warranted.

### Heterozygous and homozygous FII

Heterozygous FII was evaluated in 60,648 individuals, of whom 2,614 had the thrombophilia (Table 1). The overall OR for VTE was 2.21 (95% CI 1.70–2.87, 95% PI 0.54–9.02). Risk of primary VTE was 2.39 (95% CI 0.74–7.71) and 1.26 (95% CI 0.78–2.03) for recurrent VTE. Heterogeneity was substantial, indicated by high I^2^ and τ^2^ (Table 1). For homozygous FII, 56,260 individuals were assessed, with 27 being homozygous for FII. The overall ORfor VTE was 5.16 (95% CI 3.12–8.52, 95% PI 3.12–8.52). Risk of primary VTE was 5.46 (95% CI 2.73–10.93). Data were insufficient for calculating the risk of recurrent VTE. No heterogeneity was observed for FII homozygosity.

### Compound heterozygous FVL and FII

Compound heterozygosity was evaluated in 9,483, and 119 patients had the thrombophilia (Table 1). The overall OR for VTE was 4.64 (95% CI 2.25–9.58, 95% PI 0.15–142.6). Risk of primary VTE was 2.82 (95% CI 0.48–16.57), but data were insufficient for calculating the risk of recurrent VTE. Due to high heterogeneity for the combined effect, subgroup analysis based on study design was conducted (Table 1).

**Table 1:** Risk of venous thromboembolism

	No of studies (High Quality)	Number of VTE patients		Prediction interval (95%)	Odds Ratio (95% CI)					
		W/ thrombophilia/ Total	W/o thrombophilia/ Total		Overall effect I^2^	Primary VTE I^2^	Recurrent VTE I^2^	High quality I^2^	Case-control I^2^	Cohort I^2^
**Factor V Leiden heterozygous**	75 (18)	2,764 / 8,687	12,872 / 60,252	0.51–17.49	2.97 (2.41–3.67) *86.5*	2.74 (1.59–4.75) *41.5*	2.16 (1.16–4.03) *43.1*	2.05 (1.22–3.44) *93.0*	3.68 (2.95–4.58) *71.6*	1.56 (1.06–2.31) *93.9*
**Factor V Leiden homozygous**	65 (12)	191 / 338	13,512 / 62,384	4.61–6.74	5.58 (4.61–6.74) *0.0*	7.38 (4.68–11.65) *0.0*	3.82 (0.87–16.67) *0.0*	4.90 (3.71–6.63) *0.0*	NA	NA
**Prothrombin G20210A heterozygous**	66 (15)	929 / 2,614	12,715 / 58,034	0.54–9.02	2.21 (1.70–2.87) *65.1*	2.39 (0.74–7.71) *66.3*	1.26 (0.78–2.03) *74.2*	2.04 (1.01–4.13) *73.3*	2.37 (1.84–3.06) *58.9*	1.63 (0.37–7.23) *76.5*
**Prothrombin G20210A homozygous**	60 (12)	17 / 27	12,125 / 56,233	3.12–8.52	5.16 (3.12–8.52) *0.0*	5.46 (2.73–10.93) *0.0*	\	4.91 (2.16–11.15) *0.0*	NA	NA
**Compound heterozygous Factor V Leiden and prothrombin G20210A**	17 (4)	67 / 119	2,580 / 9,364	0.15–142.6	4.64 (2.25–9.58) *64.9*	2.82 (0.48–16.57) *0.0*	\	4.64 (2.25–9.58) *64.9*	4.42 (1.94–10.06) *66.1*	5.13 (0.16–165.63) *43.1*
**Protein C deficiency**	25 (10)	298 / 715	6,635 / 42,724	0.65–15.96	3.23 (2.05–5.08) *61.4*	2.70 (1.14–6.43) *0.0*	1.97 (0.60–6.53) *54.8*	2.21 (1.19–4.13) *35.4*	5.09 (2.79–9.28) *67.8*	2.01 (1.20–3.36) *32.4*
**Protein S deficiency**	26 (11)	436 / 1,103	6,642 / 38,570	1.09–8.36	3.01 (2.26–4.02) *42.9*	2.71 (2.14–3.43) *0.0*	1.59 (0.54–4.67) *46.8*	2.95 (1.69–5.17) *47.2*	NA	NA
**Antithrombin deficiency**	28 (12)	566 / 3,161	8,479 / 61,732	0.32–50.68	4.01 (2.50–6.44) *86.8*	2.54 (0.64–10.10) *72.0*	2.13 (1.09–4.13) *31.3*	2.81 (1.62–4.86) *88.3*	3.78 (1.93–7.41) *90.4*	3.22 (1.49–6.94) *65.3*

\ insufficient data

### PC, PS, and AT deficiency

PC was evaluated in 43,439 individuals, of whom 715 had the thrombophilia. The overall OR for VTE was 3.23 (95% CI 2.05–5.08, 95% PI 0.65–15.96). Risk of primary VTE was 2.70 (95% CI 1.14–6.43) and 1.97 (95% CI 0.60–6.53) for recurrent VTE. Substantial heterogeneity was indicated by I^2^ and τ^2^ (Table 1). For PS, 39,673 patients were evaluated, of whom 1,103 had PS deficiency. Overall OR for VTE was 3.01 (95% CI 2.26–4.02, 95% PI 1.09–8.36). Risk of primary VTE was 2.71 (95% 2.14–3.43) and 1.59 (95% 0.54–4.67) for recurrent VTE. Heterogeneity was low for PS deficiency, and subgroup analysis based on study design was not warranted. AT was evaluated in 64,893 individuals, of whom 3,161 had AT deficiency. The overall OR for VTE was 4.01 (95% CI 2.50–6.44, 95% PI 0.32–50.68). Risk of primary VTE was 2.541 (95% 0.64–10.10) and 2.13 (95% 1.09–4.13) for recurrent VTE. Heterogeneity was high for AT deficiency, except recurrent VTE.

### Publication bias

Publication bias was observed for FVL heterozygosity and PC deficiency (see supplementary information). Egger’s test was not conducted for thrombophilias with fewer than ten studies. Notably, several studies reported VTE in all individuals with thrombophilia: FVL heterozygous [14–19], FVL homozygous [15, 20–44], FII heterozygous [17–19, 27], FII homozygous [17, 24, 31, 38, 39, 45–49], compound heterozygosity [35, 37, 50–53], PC deficiency [15, 17, 39, 54, 55], PS deficiency [17, 56], and AT deficiency [17, 35, 55–57]. This raises concerns about potential selection bias. However, for FVL homozygosity, FII homozygosity, and PC deficiency, prediction intervals, heterogeneity measures, and Egger’s test indicated reliable effect estimates with little variability and no apparent bias.

## Discussion

### Summary of findings

From data encompassing 109,467 individuals, with 20% experienced VTE, adults with hereditary thrombophilia exhibit elevated VTE risk. The highest risk is associated with homozygous FVL, homozygous FII, and compound heterozygosity. In contrast, the VTE risk is lowest for FVL heterozygosity and FII heterozygosity, whereas PC, PS, and AT deficiency demonstrate an intermediate VTE risk.

### Comparison with previous systematic reviews

This study’s updated risk estimates for FVL heterozygosity and FII heterozygosity align with previous systematic reviews [58, 59]. For FVL homozygosity, we provide reliable evidence of VTE risk that is substantially lower compared to preceding work [59]. Furthermore, we did not find natural anticoagulant deficiency to be associated with high VTE risk [5, 60, 61]. This review adds to the understanding of VTE risk for individuals with hereditary thrombophilia. The discrepancies in risk estimates between the present and previous systematic reviews are presumably due to methodological differences.

### Methodological considerations

Our findings are derived from a larger population with more thrombophilic individuals compared to previous systematic reviews [58, 59]. While this enhances the precision of the risk estimates, it also introduces discrepancies. Previous studies assessing VTE risk in PC, PS, and AT deficiency often adopted a family study design. While suitable for investigating rare mutations, this approach may overestimate VTE risk for two reasons. First, yet unknown hereditary genetic variants could elevate VTE risk, and their presence is heightened in family studies [62]. Second, family studies often include paediatric individuals, and factors beyond the thrombophilia often account for the increased risk seen in paediatric populations [63]. To mitigate confounding factors from paediatric groups, we only included individuals > 15 years. Additionally, we excluded studies investigating interaction between specific comorbidities and thrombophilia. The published studies covered participants with diverse comorbidities, each potentially influencing VTE risk, presenting a realistic representation of the heterogeneous population encountered in daily clinical practice.

We included studies with venous thrombosis, irrespective of type, whereas previous studies have mainly focused on deep vein thrombosis [64] and pulmonary embolism [65]. Our approach allows for a broader evaluation of VTE risk unrestricted by thrombosis location. The association of hereditary thrombophilia with thrombosis in unusual sites is not well established [66], potentially contributing to disparities in risk estimates between our study and previous publications. Considering the population’s diversity in VTE manifestations, this review facilitates a nuanced understanding of risk evaluation. Our analyses showed minimal publication bias. Based on the prediction intervals, along with the heterogeneity estimates and NOS evaluation, there are indications that selection bias may be present in the included studies. Specifically, the overall risk estimates for FVL heterozygosity, FII heterozygosity, compound heterozygosity, PC, and AT are indicative hereof. In contrast, heterogeneity was overall low for risk estimates in the sub-analyses for VTE occurrence, and the 95% CIs were narrow, indicating their reliability.

We also excluded studies lacking explicit distinction among thrombophilia types, such as hetero- and homozygous individuals, and studies including patients with more than one thrombophilia. This ensures clarity and specificity in the risk estimates, which are essential for accurate risk stratification. For natural anticoagulant deficiency, different mutation subtypes exhibit varying thrombogenicity [67–69]. We included studies with persistent deficiency in natural anticoagulants, regardless of the mutational subtypes, since natural anticoagulant deficiency can also occur in patients without detectable mutations [70]. It is possible that the studies included in this review represent populations with less thrombogenic mutations compared to other studies. The broad prediction intervals for PC and AT deficiency reflect considerable variation in risk estimates of the individuals studies, aligning with prior findings [67, 69], that emphasise these thrombophilias should not be viewed as a homogenous group.

### Implications of the results and correlation to current guidelines

Understanding the risk of thrombosis is essential for determining the need for thromboprophylaxis and the duration of anticoagulant treatment in patients with thrombophilia. The VTE risk of the individual thrombophilia should, therefore, be taken into account along with the patient’s other risk factors. Current guidelines recommend testing asymptomatic first-degree relatives of probands with natural anticoagulant deficiency [66] and initiating thromboprophylaxis in asymptomatic individuals with PS, PC, or AT deficiency [71]. The present results do not unequivocally support this, as natural anticoagulant deficiencies showed intermediate VTE risk. However, variation in VTE risk in natural anticoagulant deficiency due to the underlying genetic subtypes needs to be considered. For thrombophilias with low VTE risk, i.e. heterozygous FVL and FII, guidelines generally suggest against testing of symptomatic or asymptomatic relatives [66, 71]. Regarding duration of anticoagulant treatment, our data showed lower risk of recurrent compared to primary VTE in all thrombophilias, except AT deficiency. The risk estimates for recurrent VTE showed greater heterogeneity than those for primary VTE, which may, in part, be due to variations in anticoagulant treatment among the included studies, where ongoing anticoagulant treatment could not be controlled for. Nevertheless, the risk of recurrent VTE was not negligible for any thrombophilia. Our data suggest that neither presence nor the type of thrombophilia should be the sole determinants of the duration of anticoagulant treatment following a VTE. Therefore, patients should, irrespective of the type of hereditary thrombophilia, be individually assessed for the risk of recurrent VTE, considering both hereditary and environmental VTE risk factors [3].

### Implications for future research

To limit prolonged anticoagulant treatment in patients with thrombophilia, future studies should evaluate the role of anticoagulant treatment in relation to the risk of recurrent VTE in these patients. Research on the phenotype of natural anticoagulant deficiency is warranted to support the present findings, particularly regarding AT deficiency. Despite no evident publication bias for FII heterozygosity or FII and FVL compound heterozygosity, our data reveals substantial heterogeneity, necessitating further research for reliable risk estimates. New studies should prioritize representativeness, comparability, and differentiation between primary and recurrent VTE. Additionally, research is essential to understand thrombophilia and thrombosis in unusual sites for better patient care.

## Conclusion

The present results highlight an increased risk of venous thromboembolism in adults with hereditary thrombophilia. However, the risk of primary VTE in patients with PC, PS, and AT deficiency is lower than previous stated. Our findings underscore significant variation in VTE risk among PC, PS, and AT deficient individuals, likely attributed to the thrombogenicity of the underlying mutation and other yet unestablished hereditary thrombophilic factors. To ensure accurate treatment for patients with hereditary thrombophilia, studies determining VTE risk for specific genotypes and providing insights to VTE risk in general are needed.

## Electronic supplementary material

Below is the link to the electronic supplementary material.

Supplementary Material 1

## Data Availability

No datasets were generated or analysed during the current study.
