# Supplementary material for: Venous thromboembolism risk in adults with hereditary thrombophilia: a systematic review and meta-analysis
Source: Ann Hematol. 2024 Aug 21;103(10):4285–94. doi: 10.1007/s00277-024-05926-2 (PMC11512919; doi:10.1007/s00277-024-05926-2)
Supplement: Supplementary file 1 — Supplementary Material 1 [file 277_2024_5926_MOESM1_ESM.docx]

**Supplementary Information**

Content

[Search strategy 2](#_Toc165970555)

[Exclusion criteria of studies 2](#_Toc165970556)

[Data extraction 3](#_Toc165970557)

[Supplementary Table 1: Descriptive characteristics of the included studies 5](#_Toc165970558)

[Studies included according to thrombophilia 18](#_Toc165970559)

[Newcastle Ottawa Scale for the included studies 19](#_Toc165970560)

[Newcastle Ottawa Score for included case-control studies 22](#_Toc165970561)

[Newcastle Ottawa Score for included cohort studies 26](#_Toc165970562)

[Forest plots 27](#_Toc165970563)

[FVL heterozygous Forest Plot 27](#_Toc165970564)

[FVL homozygous Forest Plot 33](#_Toc165970565)

[Prothrombin G20210A heterozygous Forest Plot 35](#_Toc165970566)

[Prothrombin G20210A homozygous Forest Plot 39](#_Toc165970567)

[Compound heterozygous Factor V Leiden and prothrombin G20210A Forest Plot 41](#_Toc165970568)

[PC deficiency Forest Plot 43](#_Toc165970569)

[PS deficiency Forest Plot 46](#_Toc165970570)

[AT deficiency Forest Plot 48](#_Toc165970571)

[Publication Bias 51](#_Toc165970572)

[Egger’s Regression Test 51](#_Toc165970573)

[FVL heterozygous Funnel Plot 52](#_Toc165970574)

[FVL homozygous Funnel Plot 55](#_Toc165970575)

[Prothrombin G20210A heterozygous Funnel Plot 56](#_Toc165970576)

[Prothrombin G20210A homozygous Funnel Plot 59](#_Toc165970577)

[Compound FVL and FII heterozygous Funnel Plot 60](#_Toc165970578)

[PC deficiency Funnel Plot 62](#_Toc165970579)

[PS deficiency Funnel Plot 64](#_Toc165970580)

[AT deficiency Funnel Plot 65](#_Toc165970581)

[References 67](#_Toc165970582)

## Search strategy

We searched PubMed, Embase (Ovid), and Web of Science conducted on the 17^th^ of November 2022 and the 15^th^ of November 2023:

Hereditary antithrombin deficiency OR inherited antithrombin deficiency OR hereditary protein S deficiency OR inherited protein S deficiency OR hereditary protein C deficiency OR inherited protein C deficiency OR FV Leiden OR Factor V Leiden OR prothrombin mutation OR prothrombin G20210A

| **Search results** | | |
| --- | --- | --- |
|  | Date of search | |
| Database | *17^th^ of November 2022* | *15^th^ of November 2023* |
| **PubMed** | 9,558 | 201* |
| **Embase** | 9,083 | 296** |
| **Web of Science** | 19,788 | 693*** |
| Total number of publications | 28,871 | 1,190 |

* Publication date limit: 01/11/2022 - 15/11/2023, ** Publication date limit: 2022 - 2023, *** Publication date limit: 01/11/2022 - 15/11/2023

## Exclusion criteria of studies

- Case reports, meta-analyses
- Studies where the type of hereditary thrombophilia was not distinguishable (e.g. mixed population of heterozygous and homozygous FV Leiden)
- Studies on venous thromboembolism (VTE) risk in rare subtypes of hereditary thrombophilia in comparison to other mutations (e.g. risk of VTE in SERPINC1 c.1154-2A>G vs. SERPINC1 Pro353Ala)
- Studies with no original data
- Studies where FV Leiden or FII prothrombin mutations were not genetically confirmed
- Studies that looked on genetic, but not phenotypic thrombophilia (i.e. a protein S mutation without protein S deficiency)
- Full text not retrievable
- Population < 15 years or population with undisclosed age of VTE patients
- Full text not written in either English, German, Swedish, Norwegian, or Danish
- Studies on clinical treatment of patients with hereditary thrombophilia
- Studies on risk of arterial thrombosis in hereditary thrombophilia
- Pathophysiological studies
- Technical reports (e.g. studies on laboratory methods for diagnosis of hereditary thrombophilia)
- Single family studies
- Studies on acquired thrombophilia (e.g. protein S deficiency in liver failure)
- Studies on genetically secluded populations
- Studies on contraceptives/ pregnancy/ pregnancy-related outcomes in hereditary thrombophilia
- Studies on surgery-related handling of patients with hereditary thrombophilia
- Studies set in intensive care
- Studies on specific co-morbidities in hereditary thrombophilia (e.g. cancer, heart failure, hepatic failure, angioedema etc.)
- Studies with no extractable data for VTE risk calculation

## Data extraction

| **Data extraction form** |
| --- |
| Study title |
| 1st Author |
| Year of publication |
| Country |
| Reviewer initials |
| Study design |
| **Selection of study population** |
| Consecutively |
| Random sample |
| Family study |
| Other (specify) |
| Number of VTE patients |
| Number of non-VTE patients |
| Number of individuals in risk analysis |
| Age VTE patients (mean or median) |
| Age VTE patients (Standard Deviation (SD), range or Interquartile Range (IQR)) |
| Age non-VTE patients (mean or median) |
| Age non-VTE patients (SD, range or IQR) |
| Male % VTE patients |
| Male % non-VTE patients |
| **Comorbidities in the study population? (yes/ no/ NA)** |
| Active cancer |
| Pregnancy |
| Systemic autoimmune disease |
| Arterial cardiovascular disease |
| Diabetes |
| Liver disease |
| Other comorbidities |
| Patients with comorbidities were included (yes/ no/ NA) |
| Patients with comorbidities were excluded (yes/ no/ NA) |
| Other exclusion criteria |
| **Medical treatment** |
| Anticoagulant treatment? (yes/ no/ NA) |
| Platelet-inhibitor treatment? (yes/ no/ NA)) |
| Hormonal treatment? (yes/ no /NA)) |
| **Type of VTE at outcome (n)** |
| Deep venous thrombosis (DVT) |
| Pulmonary embolism (PE) |
| Cerebral venous thrombosis (CVT) |
| Retinal vein thrombosis (RVO) |
| Abdominal vein thrombosis (splanchnic, portal, mesenteric) |
| Superficial venous thrombosis (SVT) |
| VTE type not stated |
| **Type of hereditary thrombophilia in VTE patients** |
| Number of patients with thrombophilia |
| FV Leiden heterozygous (n) |
| FV Leiden homozygous (n) |
| FII heterozygous (n) |
| FII homozygous (n) |
| FV + FII heterozygous (n) |
| Protein S deficiency (n) |
| Protein C deficiency (n) |
| Antithrombin deficiency (n) |
| **Type of hereditary thrombophilia in non-VTE patients** |
| Number of patients with thrombophilia |
| FV Leiden heterozygous (n) |
| FV Leiden homozygous (n) |
| FII heterozygous (n) |
| FII homozygous (n) |
| FV + FII heterozygous (n) |
| Protein S deficiency (n) |
| Protein C deficiency (n) |
| Antithrombin deficiency (n) |
| **Follow-up time for cohort studies** |
| **Risk of VTE (risk estimate and 95%CI)** |
| FV Leiden heterozygous |
| FV Leiden homozygous |
| FII heterozygous |
| FII homozygous |
| FV + FII heterozygous |
| Protein S deficiency |
| Protein C deficiency |
| Antithrombin deficiency |

## Supplementary Table 1: Descriptive characteristics of the included studies

| **Supplementary Table 1:Descriptive characteristics of the included studies** | | | | | | | | | | |
| --- | --- | --- | --- | --- | --- | --- | --- | --- | --- | --- |
| **Study** | **Country, Pub. year** | **VTE (total)** | **Age, yr VTE pts.** | **Age, yr non-VTE pts.** | **% male VTE pts.** | **% male non-VTE pts.** | **Comorbidities** | **Anticoagulant/ antiplatelet** | **Hormonal treatment** | **VTE type (n)** |
| **Case-control studies** | | | | | | | | | | |
| Aleksova et al (1) | Italy, 2015 | 77 (146) | 58 (SD 15) | 36 (SD 10) | 36.5 | 16.7 | Cancer, CVD | Not stated | Not stated | PE (77) |
| Alfeel et al (2) | Sudan, 2020 | 126 (231) | 43.2 (ran 40 - 70) | 41.1 (ran 40 - 70) | 51.6 | 60.7 | Cancer, CVD, diabetes, hypertension, renal disease | Not stated | Not stated | DVT (126) |
| Alhenc-Gelas et al (3) | France, 1999 | 205 (603) | 42.2 (SD 11.1) | 42.9 (SD 9.5) | 44.8 | 50.0 | Cancer, CVD | Not stated | Yes | PE (20), DVT (185) |
| Almawi et al (4) | Lebanon, 2005 | 198 (895) | 38.2 (SD 11.4) | 33.4 (SD 11.8) | 42.0 | 43.0 | Not stated | Not stated | Not stated | DVT (198) |
| Altinisik et al (5) | Turkey, 2008 | 50 (75) | 48.14 (ran 19 - 78) | 32.4 (ran 22 - 47) | 56.0 | 40.0 | Not stated | Not stated | Not stated | DVT (12), PE (7), CVT (31) |
| Aras et al (6) | Turkey, 2001 | 40 (90) | 59 (ran 35 - 77) | 55 (ran (34 - 76) | 52.5 | 52.0 | Diabetes, hypertension, dyslipidaemia | Not stated | Not stated | RVO (40) |
| Arsène et al (7) | France, 2005 | 234 (414) | 62 (ran 17 - 87) | 32 (ran 18 - 85) | 63.7 | 34.4 | GCA, hypertension, glaucoma, hyperlipidaemia, obesity | Not stated | Not stated | RVO (234) |
| Arsov et al (8) | Macedonia, 2006 | 190 (390) | 42.5 (SD 14.5) | Not stated | 40.0 | 40.0 | Pregnancy, trauma, surgery | Not stated | Not stated | DVT (190) |
| Ates et al (9) | Turkey, 2006 | 54 (104) | Ran 22 - 86 | Ran 24 - 78 | Not stated | Not stated | Not applicable | No/ not stated | No | RVO (54) |
| Ben Salem-Berrabah et al (10) | Tunisia, 2012 | 26 (223) | 38.3 (SD 14.5) | 31.0 (SD 9.6) | 19.2 | 63.9 | Not stated | Not stated | Not stated | CVT (26) |
| Beye et al (11) | Germany, 2017 | 101 (202) | 38.8 (SD 12.8) | 39.2 (SD 12.2) | 23.7 | 23.7 | Not stated | Not stated | Not stated | CVT (101) |
| Bezgin et al (12) | Turkey, 2018 | 310 (599) | 52.3 (SD 16.9) | 49.7 (SD 14.7) | 50.3 | 56.4 | Cancer, pregnancy, AD, immobilisation | No/ not stated | Yes | DVT (247), PE (20), PE + DVT (43) |
| Blom et al (13) | Netherlands, 2005 | 144 (2,162) | 45.1 (ran 20 - 67) | 50.2 (ran 28 - 66) | 44.1 | 49.9 | Cancer, CVD, CVC, pregnancy, surgery, immobilisation, obesity | No/ not stated | Yes | DVT (144) |
| Bombeli et al (14) | Switzerland, 2002 | 552 (672) | 45.9 (ran 17 - 77) | 37.4 (ran 19 - 62) | 44.5 | 40 | Cancer, pregnancy, diabetes, liver disease, hypertension, surgery, trauma, immobilisation, infection | Yes/ not stated | Yes | DVT (391), CVT (51), RVO (68), PVT (42) |
| Bouaziz-Borgi et al (15) | Tunisia, 2007 | 126 (323) | 38.2 (SD 11.4) | Not stated | Not stated | Not stated | Not stated | Not stated | Not stated | DVT (126) |
| Boyanovsky et al (16) | Bulgaria, 2001 | 120 (220) | 44.8 (ran 21 - 68) | Not stated | 35.0 | Not stated | Not applicable | Not stated | Not stated | DVT (37), PE (75), DVT+PE (8) |
| Cernera et al (17) | Italy, 2020 | 126 (556) | 53 (ran 16 - 79) | 43 (ran 5 - 85) | 35.8 | 38.4 | Not stated | Yes/ not stated | Not stated | SVT (126) |
| Chen et al (18) | Taiwan, 2002 | 54 (154) | 43 (ran 18 - 65) | Not stated | 53.5 | Not stated | Pregnancy | No/ not stated | Yes | DVT (25), CVT (5), VTE (34) |
| Coen et al (19) | Croatia, 2001 | 160 (315) | 39 (ran 16 - 84) | Ran 15 - 70 | 35.6 | 29.0 | Not stated | Not stated | Not stated | DVT (138), PE (7), SVT (12), VTE (3) |
| Cumming et al (20) | United Kingdom, 1997 | 166 (330) | 43 (ran 17 - 88) | 34 (ran 18 - 64) | Not stated | Not stated | Not applicable | Not stated | Not stated | VTE (166) |
| Daraban et al (21) | Romania, 2016 | 29 (160) | 30.1 (SD 8.2) | 37.4 (SD 7.6) | 0 | 0 | Cancer, pregnancy, CVD, diabetes, hypertension, dyslipidaemia, obesity | Not stated | Yes | VTE (29) |
| De Moerloose (22) | Switzerland, 2000 | 172 (738) | Ran 21 - 99 | 60 (ran 19 – 97) | 45.3 | 43.1 | Not stated | Not stated | Not stated | DVT (83), PE (99) |
| de Paula Sabino et al (23) | Brazil, 2007 | 275 (599) | 35.5 (SD 13.7) | 36.2 (SD 13.5) | 19.8 | 28.7 | Pregnancy, AD, sepsis, immobilisation, surgery | Not stated | Yes | DVT (157), PE (35), CVT (17), RVO (11), PVT (15), SVT (15), VTE (25) |
| de Visser et al (24) | Netherlands, 2000 | 461 (933) | 47 (ran 16 - 70) | 47 (ran 16 - 73) | 33.0 | 33.0 | Not stated | Not stated | Not stated | DVT (461) |
| Delahousse et al (25) | France, 1998 | 83 (143) | 62 (ran 17 - 88) | 38 (ran (23 - 85) | 63.8 | 45.0 | Not stated | Not stated | Not stated | RVO (83) |
| Delluc et al (26) | France, 2010 | 677 (1,354) | 67 (SD 17) | 68 (SD 17) | 44.0 | 44.0 | Not applicable | Not stated | Not stated | DVT (292), PE (129), DVT+PE (256) |
| Di Minno et al (27) | Italy, 2013 | 601 (2,274) | 48.6 (SD 14.5) | 49.2 (SD 10.9) | 42.1 | 39.6 | Surgery, trauma, hypertension, obesity | Not stated | No | VTE (601) |
| Dimri et al (28) | India, 2019 | 150 (300) | Ran 18 - 60 | Not stated | 81.0 | 81.0 | Not applicable | No/ not stated | No | DVT (87), PE (11), CVT (52) |
| Djordjevic et al (29) | Serbia, 2004 | 175 (295) | 25.3 | 38.2 | 41.7 | 70.0 | Not stated | Not stated | Not stated | DVT (113), VTE (62) |
| Farajzadeh et al (30) | Iran, 2014 | 193 (693) | 46.2 (SD 4.7) | 46.3 (SD 5.8) | 53.3 | 55.0 | Not applicable | Not stated | Not stated | DVT (193) |
| Folsom et al (31) | USA, 2002 | 231 (720) | 64 (ran 45 - 94) | 64 (ran 45 - 94) | 47.0 | 47.0 | Not stated | Not stated | Not stated | VTE (231) |
| Gorski et al (32) | Italy, 2018 | 171 (469) | 38 (SD 13) | 43 (SD 14) | 25.7 | 40.2 | Pregnancy, AD, infection | Not stated | Yes | CVT (171) |
| Heijboer et al (33) | Netherlands, 1990 | 277 (415) | 56 (ran 17 - 91) | 57 (ran (18 - 90) | 50.0 | 50.0 | Not applicable | Not stated | Not stated | DVT (277) |
| Hillarp et al (34) | Sweden, 1997 | 99 (282) | 64 (ran 21 - 89) | 59 (ran 34 - 86) | 41.4 | 63.8 | Not stated | Not stated | Not stated | DVT (99) |
| Jackson et al (35) | United Kingdom, 2000 | 517 (995) | 41 (ran 18 - 65) | 38 (ran 18 - 65) | 30.9 | Not stated | Not stated | Not stated | Not stated | DVT (319), PE (198) |
| Jusić-Karić et al (36) | Bosnia and Herzegovina, 2016 | 111 (318) | 53 (ran 21 - 84) | 45 (ran 18 - 84) | 46.8 | 49.2 | Not stated | Not stated | Not stated | DVT (111) |
| Karasu et al (37) | Netherlands, 2016 | 394 (820) | 78.7 (ran 70 - 101) | 77.5 (ran 70 - 96) | 41.4 | 48.5 | Not applicable | Yes/ not stated | Not stated | VTE (394) |
| Kalayci et al (38) | Turkey, 1999 | 52 (133) | 60.5 (ran 25 - 83) | Not stated | 59.5 | Not stated | Cancer, AD, diabetes, glaucoma, hypertension, renal disease, anaemia, hyperlipidaemia | Not stated | Not stated | RVO (52) |
| Keijzer et al (39) | Netherlands, 2002 | 171 (632) | 61 (ran 23 - 87) | 51 (ran 21 - 81) | 52.0 | 42.0 | Not applicable | Yes/ not stated | Not stated | VTE (171) |
| Kupeli et al (40) | Turkey, 2011 | 80 (177) | 54.4 (ran 17 - 40) | Not stated | 55.0 | Not stated | Not applicable | No/ not stated | Yes | PE (51), DVT + PE (29) |
| Legnani et al (41) | Italy, 2002 | 301 (951) | 37 (ran 16 - 58) | 33 (ran 15 - 49) | 0 | 0 | Pregnancy, surgery, trauma, immobilisation, fracture | No/ not stated | Yes | DVT (241), DVT+PE (60) |
| Lichy et al (42) | Germany, 2006 | 77 (279) | 38.0 (SD 19.5) | 26.5 (SD (15.5) | 22.1 | 41.8 | Pregnancy, AD, infection | Not stated | Yes | CVT (77) |
| Lijfering et al (43) | Netherlands, 2010 | 325 (646) | 36 (SD 15) | 39 (SD 16) | 40.0 | 33.0 | Cancer, pregnancy, surgery, trauma, immobilisation | Yes/ not stated | Yes | VTE (325) |
| Lindmarker et al (44) | Sweden, 1999 | 65 (467) | 62 (IQR 49 - 68) | 57 (not stated) | 67.4 | Not stated | Not stated | Yes/ not stated | Not stated | VTE (65) |
| Linna et al (45) | Finland, 1997 | 46 (188) | 40.5 (ran 15 - 50) | 46 (ran 22 - 66) | 52.0 | 56.0 | Not stated | Not stated | Not stated | RVO (46) |
| Mansilha et al (46) | Portugal, 2002 | 40 (140) | 27 (ran 17 - 40) | Not stated | 35.0 | Not stated | Not stated | Not stated | Not stated | VTE (40) |
| Mansilha et al (47) | Portugal, 2006 | 99 (199) | 27 (ran 16 - 40) | 27 (ran 16 - 40) | 31.5 | 31.5 | Not stated | Not stated | Not stated | DVT (99) |
| Manten et al (48) | Netherlands, 2006 | 256 (730) | 42.7 (not stated) | 44.1 (not stated) | 48.3 | 42.6 | Pregnancy, surgery, immobilisation, trauma | Yes/ not stated | Not stated | DVT (210), PE (45) |
| Marcucci et al (49) | Italy, 2001 | 100 (200) | 59 (ran 18 - 77) | 56 (ran 18 - 84 | 54.0 | 58.0 | CVD, diabetes, hypertension, hyperlipidaemia | Not stated | Not stated | CVT (100) |
| Marcucci et al (50) | Italy, 2000 | 53 (106) | 59 (ran 18 - 77) | 57 (ran 22 - 84) | 54.7 | 52.8 | CVD, diabetes, hypertension, hyperlipidaemia | Not stated | Not stated | RVO (53) |
| Mitsuguro et al (51) | Japan, 2010 | 108 (4,625) | 50.5 (ran 15 - 89) | Not stated | 46.0 | Not stated | Diabetes, hypertension, hyperlipidaemia | Not stated | Not stated | VTE (108) |
| Nizankowska-Mogilnicka et al (52) | Poland, 2003 | 149 (249) | 47.9 (ran 19 - 86) | 45.4 (ran 19 - 61) | 61.0 | 60.0 | Obesity, immobilisation | Not stated | Not stated | DVT (33), PE (48), DVT+PE (68) |
| Obeid et al (53) | Syria, 2003 | 78 (176) | 33 (SD 10) | 44 (SD 10) | 57.0 | 55.0 | Pregnancy, obesity, surgery, infection | Yes/ Yes | Yes | VTE (78) |
| Okumus et al (54) | Turkey, 2008 | 191 (382) | 53.3 (ran 16 - 88) | 50.6 (ran 16 - 88) | 45.5 | 45.5 | Cancer, pregnancy, CVD, immobilisation, obesity, trauma, kidney disease | Not stated | Yes | DVT (37), PE (80), DVT+PE (74) |
| Pérez-Ceballos et al (55) | Span, 2002 | 204 (408) | 52.9 (SD 18.7) | 53.2 (SD 17.8) | 53.4 | 53.4 | Not stated | Not stated | Not stated | DVT (204) |
| Pestana et al (56) | Venezuela, 2009 | 208 (342) | 40.1 (SD 15.4) | 38.4 (SD 15.1) | 19.2 | 39.2 | Diabetes, hypertension | Not stated | Yes | DVT (208) |
| Primignani et al (57) | Italy, 2005 | 565 (1,265) | 39 (ran 15 - 66) | 44 (ran 12 - 84) | 36.9 | 44.8 | Cancer, pregnancy, AD, surgery, trauma, immobilisation | Yes/ not stated | Yes | VTE (565) |
| Rahimi et al (58) | Iran, 2010 | 80 (180) | 42.1 (SD 13.0) | 37.6 (SD 13.3) | 45.0 | 45.0 | Cancer, pregnancy, immobilisation | Not stated | Yes | DVT (80) |
| Renner et al (59) | Austria, 2000 | 154 (462) | 55 (ran 17 - 90) | 54 (ran 17 - 91) | 39.0 | 39.0 | Cancer, AD | Not stated | Not stated | DVT (117), DVT+PE (37) |
| Ridker et al (60) | USA, 1999 | 218 (1,992) | 59.9 (SD 9.0) | 59.1 (SD 8.6) | 100 | 100 | Cancer, CVD, diabetes, surgery, hypertension, hyperlipidaemia, trauma | Not stated/ Yes | No | VTE (218) |
| Rosendaal et al (61) | Netherlands, 1995 | 471 (945) | 40.3 (ran 15 - 59) | Not stated | 34.3 | Not stated | Pregnancy, surgery, immobilisation | Yes/ not stated | Yes | DVT (471) |
| Russo et al (62) | Italy, 2005 | 113 (217) | Ran 18 - 77 | Ran 18 - 65 | 50.4 | 72.1 | Not stated | Not stated | Not stated | RVO (113) |
| Sakata et al (63) | Japan, 2004 | 108 (4,625) | 53.7 (SD 17.6) | Ran 32 - 89 | 50.0 | 46.2 | Not stated | Not stated | Not stated | DVT (108) |
| Salazar-Sanchez et al (64) | Costa-Rica, 2007 | 120 (253) | 35.8 (SD 15.5) | 36.9 (SD 16.6) | 29.2 | 36.1 | Diabetes, obesity, hypertension, hyperlipidaemia | Not stated | Yes | DVT (47), PE (49), SVT (8), VTE (16) |
| Salomon et al (65) | Israel, 1998 | 102 (207) | 64.6 (SD 12.5) | 57.8 (SD 18.8) | 61.9 | 56.5 | CVD, diabetes, hyperlipidaemia | Not stated | Not stated | RVO (102) |
| Shen et al (66) | Taiwan, 2000 | 116 (241) | 47.5 (ran 17 - 80) | 45.5 (ran 14 - 83) | 50.0 | 53.6 | Not applicable | Not stated | Not stated | VTE (116) |
| Souto et al (67) | Spain, 1998 | 116 (317) | 47.8 (ran 16 - 83) | 40.0 (ran 18 - 74) | 43.9 | 65.2 | Cancer, pregnancy | Not stated | Not stated | VTE (116) |
| Svensson et al (68) | Sweden, 1997 | 223 (511) | 63 (ran 20 - 89) | 59 (ran 34 - 86) | 40.3 | 63.1 | Not stated | Not stated | Not stated | DVT (223) |
| Tosetto et al (69) | Italy, 1999 | 111 (335) | 54 (ran 18 - 65) | 54 (ran 18 - 65) | 37.9 | 37.9 | Not stated | Yes/ not stated | Yes | VTE (111) |
| Tony et al (70) | India, 2023 | 147 (297) | 43 (ran 21 - 80) | Not stated | 62.6 | Not stated | Cancer, pregnancy, diabetes, hypertension, dyslipidaemia, obesity, immobilisation, trauma, surgery | Yes/ not stated | Not stated | DVT (116), DVT+PE (31) |
| Trégouët et al (71) | France, 2009 | 453 (1681) | 44 (SD 14) | 47 (SD 13) | 30.0 | 48.0 | Pregnancy, surgery, immobilisation | Not stated | Yes | VTE (453) |
| Weger et al (72) | Austria, 2005 | 294 (588) | 67 (ran 22 - 93) | 67 (ran 22 - 92) | 43.5 | 43.5 | Diabetes, hypertension, hyperlipidaemia | Not stated | Not stated | RVO (294) |
| Weih et al (73) | Germany, 1998 | 12 (199) | 33.8 (ran 21 - 60) | Not stated | 16.6 | Not stated | Pregnancy | Not stated | Yes | CVT (12) |
| Zalavras Ch et al (74) | Greece, 2003 | 176 (476) | 45 (ran 18 - 69) | 35 (ran 18 - 60) | 45.4 | 77.6 | Not stated | Not stated | Not stated | DVT (176) |
| Zerjavic et al (75) | Slovenia, 2010 | 444 (569) | 50 (ran 19 - 85) | 49 (ran 30 - 73) | 40.7 | 56.0 | CVD | Not stated | Not stated | VTE (444) |
| Zhang et al (76) | China, 2018 | 348 (746) | 55.9 (SD 13.2) | 56.8 (SD 11.9) | 51.0 | 51.0 | Diabetes, hypertension | Yes/ not stated | Yes | DVT (348) |
| **Cohort Studies** | | | | | | | | | | |
| Brouwer et al (77) | Netherlands, 2005 | 45 (424) | > 15 | > 15 | Not discernible | Not discernible | Pregnancy, CVD, diabetes, hypertension, hyperlipidaemia | Yes/ not stated | Yes | DVT (30), PE (7), VTE (8) |
| Castaman et al (78) | Italy, 1999 | 8 (36) | 53.5 | 47.0 | Not stated | Not stated | CVD | Not stated | Not stated | VTE (8) |
| Cohen et al (79) | France, 2012 | 87 (1,074) | 36.4 (SD 12.0) | 32.3 (SD 18.0) | Not discernible | 40.0 | Not stated | Yes/ not stated | Yes | VTE (87) |
| Coppens et al (80) | Netherlands, 2006 | 4 (464) | Ran 18 - 60 | Ran 15 - 87 | 75.0 | Not discernible | Cancer, pregnancy, AD, CVD, diabetes, hypertension | Yes/ not stated | Yes | DVT (2), PVT (1), PE (1) |
| Couturaud et al (81) | France, 2006 | 70 (553) | > 16 | > 16 | Not discernible | Not discernible | Cancer, pregnancy | Yes/ not stated | Yes | VTE (70) |
| De Stefano et al (82) | Italy, 1999 | 120 (395) | Ran 15 - 69 | Ran 6 - 72 | Not discernible | Not discernible | Pregnancy, surgery, trauma, immobilisation | No/ not stated | Yes | VTE (120) |
| De Stefano et al (83) | Italy, 2001 | 105 (335) | Ran 17 - 64 | Ran 6 - 72 | Not discernible | Not discernible | Pregnancy, surgery, trauma, immobilisation | Not stated | Yes | VTE (105) |
| Di Minno et al (84) | Italy, 2014 | 160 (571) | 35.0 (SD 12.7) | 48.3 (SD 14.7) | 59.5 | 41.9 | Not applicable | Yes/ not stated | No | VTE (160) |
| Eichinger et al (85) | Austria, 2002 | 61 (287) | 50.0 (SD 17.5)* | 50.0 (SD 17.5)* | Not discernible | Not discernible | Not applicable | Not stated | Not stated | PE (21), VTE (40) |
| Evensen et al (86) | Norway, 2021 | 1,493 (14,562) | 61 (SD 15) | 51 (SD 17) | 47.1 | 47.1 | Cancer, pregnancy, CVD, diabetes, surgery, trauma, immobilisation | Not stated | Yes | DVT (790), PE (506),VTE (197) |
| Hodeib et al (87) | Egypt, 2021 | 58 (224) | 52.1 (SD 6.3) | 51.6 (SD 6.0) | 48.3 | 48.2 | Not applicable | No/ not stated | No | 58 (VTE) |
| Lijfering et al (88) | Netherlands, 2009 | 62 (396) | Ran 15 - 69* | Ran 15 - 69* | 65.9* | 65.9* | Not stated | No/ not stated | Not stated | 62 (DVT) |
| Mahmoodi et al (89) | Netherlands, 2010 | 27 (335) | Ran 18 - 52* | Ran 18 - 52* | 49.2* | 49.2* | Cancer, pregnancy, surgery, trauma, immobilisation | Yes/ not stated | Yes | VTE (27) |
| Makris et al (90) | United Kingdom, 2000 | 24 (96) | Ran 17.1 - 69.5* | Ran 17.1 - 69.5* | 41.6* | 41.6* | Pregnancy, trauma, surgery, immobilisation | Yes/ not stated | Yes | VTE (24) |
| Manderstedt et al (91) | Sweden, 2022 | 3,177 (28,794) | 59.7 (SD 7.4) | 57.8 (SD 7.6) | 39.6 | 41.2 | Hypertension | Not stated | Not stated | VTE (3,177) |
| Mateo et al (92) | Spain, 1998 | 150 (583) | 44.7 (SD 18.8)* | 44.7 (SD 18.8)* | 42.0* | 42.0* | Cancer, pregnancy, varicose veins, immobilisation, obesity, surgery | Yes/ not stated | Yes | VTE (150) |
| Méan et al (93) | Switzerland, 2023 | 39 (240) | 74 (IQR 69 - 79)* | 74 (IQR 69 - 79)* | 53.0* | 53.0* | Cancer, CVD, diabetes, infection, hypertension, obesity | No/ not stated | Not stated | VTE (39) |
| Middeldorp et al (94) | Netherland, 2001 | 9 (470) | 44 (ran 15 - 95) | 43 (ran 16 -89) | 46.0 | 50.0 | Pregnancy, surgery, trauma | Yes/ not stated | Yes | VTE (9) |
| Miles et al (95) | USA, 2001 | 29 (218) | Ran 40 - 84 | Ran 40 - 84 | 100 | 100 | Not stated | Not stated/ Yes | Not applicable | VTE (29) |
| Olivo Freites et al (96) | USA, 2022 | 124 (528) | > 18* | > 18* | 48.7* | 48.7* | Not applicable | No/ not stated | No | VTE (124) |
| Pires et al (97) | Brazil, 2019 | 13 (189) | 30.8 (IQR 24.7 - 40.9)* | 30.8 (IQR 24.7 - 40.9)* | 86.2* | 86.2* | Cancer, pregnancy, AD, liver disease, nephrotic syndrome, surgery, CVD | No/ not stated | Yes | CVT (13) |
| Puhr et al (98) | Austria, 2020 | 312 (640) | 48.6 (IQR 37.2 - 61.5)* | 48.6 (IQR 37.2 - 61.5)* | 46.0* | 46.0* | Not applicable | No/ not stated | Yes | DVT (177), PE (135) |
| Ridker et al (99) | USA, 1995 | 11 (77) | Ran 40 - 84 | Ran 40 - 84 | 100 | 100 | Not applicable | No/ Yes | No | VTE (11) |
| Satpanich et al (100) | Thailand, 2019 | 22 (198) | 53.4 (SD 19.6) | 63.8 (SD 17.3) | 31.8 | 31.8 | AD, CVD, diabetes, hypertension, chronic kidney disease, HIV, immobilisation, surgery | Yes/ No | Yes | VTE (22) |
| Simioni et al (101) | Italy, 1997 | 49 (251) | Ran 23 - 84* | Ran 23 - 84* | 52.2* | 52.2* | Pregnancy, trauma, surgery, immobilisation | Yes/ not stated | Yes | VTE (49) |
| Simioni et al (102) | Italy, 2002 | 10 (561) | Ran 16 - 93* | Ran 16 - 93* | 48.1* | 48.1* | Pregnancy, immobilisation, trauma | Yes/ not stated | Yes | VTE (10) |
| Tirado et al (103) | Spain, 2001 | 140 (584) | 30.2 (SD 14.6)* | 30.2 (SD 14.6)* | Not discernible | Not discernible | Cancer, pregnancy, CVD, varicose veins, surgery, immobilisation | No/ not stated | Yes | VTE (140) |
| Tormene et al (104) | Italy, 2004 | 8 (294) | 44 (ran 15 - 87)* | 44 (ran 15 - 87)* | 44.0* | 44.0* | Pregnancy, trauma, surgery, immobilisation | Not stated | Yes | DVT (7), DVT+PE (1) |
| van Boven et al (105) | Netherlands, 1999 | 13 (86) | 43 (ran 15 - 88) | 44 (ran 18 - 86) | 41.3 | 52.2 | Pregnancy, surgery, immobilisation | Yes/ not stated | Yes | VTE (13) |
| Weingarz et al (106) | Germany, 2015 | 261 (1,221) | 43 (SD 17) | Not discernible | 41.9 | Not discernible | Cancer, pregnancy, surgery, immobilisation, inflammation | No/ not stated | Yes | DVT (160), PE (71), VTE (30) |
| Zöller et al (107) | Sweden, 2018 | 220 (5,096) | 56 (SD 6)* | 56 (SD 6)* | 42.5* | 42.5* | CVD, diabetes, hypertension, hyperlipidaemia | Not stated | Yes | VTE (220) |

Abbreviations Table 1: ran: range, CVD: cardiovascular disease, CVT: cerebral venous thrombosis, DVT: deep venous thrombosis, PE: pulmonary embolism, SD: standard deviation, yr: year, RVO: retinal vein occlusion, GCA: giant cell arteritis, AD: autoimmune disease, CVC: central venous catheter, PVT: portal vein thrombosis, SVT: superficial venous thrombosis, * overall population

## Studies included according to thrombophilia

- FVL heterozygous: 57 case-control (1-8, 10-13, 15-17, 19, 21-26, 29, 30, 32, 35-45, 47-50, 52-59, 61, 62, 64, 68, 70-73, 76) and 18 cohort studies (78, 81, 82, 85, 87, 88, 91, 94-99, 101-103, 106, 107)
- FVL homozygous: 52 case-control (1-6, 8, 10-13, 15-17, 19, 21-26, 29, 30, 35-42, 44, 45, 47, 48, 50, 52-59, 61, 62, 64, 68, 71-73, 76) and 13 cohort studies (78, 81, 82, 87, 91, 94-97, 99, 101-103, 107)
- FII heterozygous: 53 case-control (1-5, 7, 10-13, 16, 17, 19-21, 23, 25, 26, 29-32, 34, 36-44, 46, 47, 49, 50, 52, 54, 55, 57-60, 62, 64, 65, 67, 69-72, 74, 75) and 13 cohort studies (78, 80, 81, 83, 87, 91, 95-98, 102, 104, 106)
- FII homozygous: 49 case-control (1-5, 7, 10-13, 16, 17, 19-21, 23, 25, 26, 29-32, 34, 36-42, 44, 47, 50, 52, 54, 55, 57-60, 62, 64, 65, 67, 69, 71, 72, 74, 75) and eleven cohort studies (78, 80, 81, 83, 87, 91, 95-97, 102, 104)
- FV/FII compound heterozygous: 14 case-control (4, 11, 13, 19, 25, 31, 32, 41, 43, 47, 52, 54, 59, 69) and three cohort studies (81, 95, 103)
- PC deficiency: 16 case-control (5, 9, 14, 18, 25, 28, 32, 33, 43, 50, 52, 54, 57, 63, 66, 79) and nine cohort studies (78, 89, 91-93, 96, 100, 103, 106)
- PS deficiency: 15 case-control (108) and eleven cohort studies (77, 78, 89-93, 96, 100, 103, 106)
- AT deficiency: 17 case-control (9, 14, 18, 25, 27, 28, 32, 33, 43, 50-52, 57, 63, 66, 79) and eleven cohort studies (78, 84, 86, 89, 91-93, 96, 103, 105, 106)

| Newcastle Ottawa Scale for the included studies |
| --- |
| Case-control study |
| **Selection – a maximum of one star can be given for each numbered item** |
| 1) Is the case definition adequate? |
| a) yes, with independent validation* |
| *How were the VTE patients diagnosed?* |
| *PE: ventilation-perfusion (VQ) or CT-pulmonary angiography (CTPA) scan [1]* |
| *DVT: ultrasound/ duplex ultrasound [1]* |
| *CVT: MR venography, digital subtraction angiography, CT venography [2]* |
| *RVO: Optical coherence tomography (OCT), fluorescein angiography (FA)/ OCT-angiography [3]* |
| *Splanchnic VTE: Doppler ultrasonography, angiography, CT/ MR angiography[4,5]* |
| b) yes, e.g. record linkage or based on self-reports |
| *Record linkage (e.g. ICD codes in database) or self-report with no reference to primary record* |
| c) no description |
| 2) Representativeness of the cases |
| a) consecutive or obviously representative series of cases* |
| *All eligible patients with VTE over a defined period of time, OR all VTE patients. in a defined catchment area, OR all cases in a defined hospital, clinic, group of hospitals, or health maintenance organisation, OR an appropriate sample of those cases (e.g. random sample)* |
| b) potential for selection biases or not stated |
| *Not satisfying requirements in part (a), or not stated* |
| *3) Selection of Controls* |
| a) community controls* |
| *Community controls (i.e. same community as cases)* |
| b) hospital controls/ non-representative controls |
| *Hospital controls, within same community as cases (i.e. not another city) but derived from a hospitalised population OR controls come from a different community than the cases* |
| c) no description |
| 4) Definition of Controls |
| a) no history of VTE* |
| *For primary VTE: no history of disease, for recurrent VTE: a minimum of one verified VTE* |
| b) no description of source |
| *No mention of history of VTE* |
| **Comparability – a maximum of two stars can be given** |
| 1) Comparability of cases and controls on the basis of the design or analysis |
| a) study controls for a major additional VTE risk factor?* |
| *Does the study control for e.g. age, immobilisation, comorbidities (e.g. active cancer), pregnancy, oral contraceptive use, surgery or gender as appropriate in the setting* |
| b) study controls for additional factors * |
| *Does the study control for more than one additional VTE risk factor?* |
| c) the study does not control for any/ additional VTE risk factors |
| **Exposure – a maximum of one star can be given for each numbered item** |
| 1) Ascertainment of exposure |
| a) secure record * |
| *Secure diagnosis of thrombophilia. I.e. PCR diagnosis of FV Leiden G1691A and FII G20210A prothrombin mutation, repeated measurement of low PC, PS, or antithrombin levels OR genetic manifestation of mutation in the PROC, PROS1, or SERPINC1 gene.* |
| b) structured interview where blind to case/control status* |
| c) interview not blinded to case/control status |
| d) written self-report or medical record only |
| *I.e. the study relied on previous records for thrombophilia definition e.g. ICD-codes.* |
| e) no description |
| *How thrombophilia status was defined not stated.* |
| 2) Same method of ascertainment for cases and controls |
| a) yes* |
| *Testing for thrombophilia was conducted in all participants.* |
| b) no |
| *Thrombophilia was only established in some participants.* |
|  |
| Cohort study |
| **Selection - a maximum of one star can be given for each numbered item** |
| 1) Representativeness of the exposed cohort (thrombophilia) |
| a) truly representative of the average adult (>15 yrs) thrombophilia patient in the community* |
| *All patients or random sampling of study population.* |
| b) somewhat representative of the average adult (>15 yrs) thrombophilia patient or background in the community * |
| *Non-random sampling of study population.* |
| c) selected group e.g. nurses, volunteers |
| *Specific population, not representative of the whole community.* |
| d) no description of the derivation of the cohort |
| 2) Selection of the non-exposed cohort |
| a) drawn from the same community as the exposed cohort * |
| *Participants without thrombophilia came from the same community as the participants with thrombophilia.* |
| b) drawn from a different source |
| *Participants without thrombophilia were selected from a different community.* |
| c) no description of the derivation of the non-exposed cohort |
| *Not defined how non-thrombophilic patients were included.* |
| 3) Ascertainment of exposure |
| a) secure record * |
| *Secure diagnosis of thrombophilia. I.e. PCR diagnosis of FV Leiden G1691A and FII G20210A prothrombin mutation, measurement of low PC, PS, or antithrombin levels OR genetic manifestation of mutation in the PROC, PROS1, or SERPINC1 gene.* |
| b) structured interview * |
| *I.e. the study relied on previous records for thrombophilia definition e.g. ICD-codes.* |
| c) written self-report |
| *Participants’ own information regarding thrombophilia was used.* |
| d) no description |
| 4) Demonstration that outcome of interest was not present at start of study |
| a) yes * |
| *I.e. for primary thrombosis, no history of VTE, for recurrent thrombosis, verified 1st thrombosis record* |
| b) no |
| **Comparability - a maximum of two stars can be given** |
| 1) Comparability of cohorts on the basis of the design or analysis |
| a) study controls for a major additional VTE risk factor?* |
| *Does the study control for e.g. age, immobilisation, comorbidities (e.g. active cancer), pregnancy, oral contraceptive use, surgery or gender as appropriate in the setting* |
| b) study controls for additional factors * |
| *Does the study control for more than one additional VTE risk factor?* |
| c) study does not control for VTE risk factors |
| **Outcome - a maximum of one star can be given for each numbered item** |
| 1) Assessment of outcome |
| a) independent blind assessment* |
| *PE: ventilation-perfusion (VQ) or CT-pulmonary angiography (CTPA) scan* |
| *DVT: ultrasound/ duplex ultrasound* |
| *CVT: MR venography, digital subtraction angiography, CT venography* |
| *RVO: OCT, FA/ OCTA* |
| *Splanchnic VTE: doppler ultrasonography, angiography, CT/ MR angiography* |
| b) record linkage* |
| *Record linkage (e.g. ICD codes in database or medical record)* |
| c) self-report |
| *Participants’ own record of VTE* |
| d) no description |
| 2) Was follow-up long enough for outcomes to occur |
| a) yes* |
| b) no |
| 3) Adequacy of follow up of cohorts |
| a) complete follow up - all subjects accounted for * |
| b) subjects lost to follow up unlikely to introduce bias - small number lost (< 5 % follow up, or description provided of those lost)* |
| c) follow up rate > 5% and no description of those lost |
| d) no statement |

| Newcastle Ottawa Score for included case-control studies Median score = 5 | | | | | |
| --- | --- | --- | --- | --- | --- |
| **Study** | **Selection** (max. four stars) | **Comparability** (max. two stars) | **Exposure** (max. two stars) | **Overall score** (max. 8 stars) | **Evaluation** |
| Aleksova et al (1) | 4 | 0 | 2 | 6 | Intermediate risk of bias |
| Alfeel et al (109) | 4 | 0 | 2 | 6 | Intermediate risk of bias |
| Alhenc-Gelas et al (3) | 3 | 2 | 2 | 7 | Low risk of bias |
| Almawi et al (4) | 1 | 0 | 2 | 3 | High risk of bias |
| Altinisik et al (5) | 0 | 0 | 2 | 2 | High risk of bias |
| Aras et al (6) | 3 | 0 | 2 | 5 | Intermediate risk of bias |
| Arsène et al (7) | 3 | 0 | 2 | 5 | Intermediate risk of bias |
| Arsov et al (8) | 2 | 0 | 2 | 4 | Intermediate risk of bias |
| Ates et al (9) | 1 | 2 | 1 | 4 | Intermediate risk of bias |
| Ben Salem-Berrabah et al (10) | 1 | 0 | 2 | 3 | High risk of bias |
| Beye et al (11) | 3 | 0 | 2 | 5 | Intermediate risk of bias |
| Bezgin et al (110) | 2 | 1 | 2 | 5 | Intermediate risk of bias |
| Blom et al (13) | 3 | 0 | 2 | 5 | Intermediate risk of bias |
| Bombeli et al (14) | 3 | 0 | 2 | 5 | Intermediate risk of bias |
| Bouaziz-Borgi et al (15) | 1 | 0 | 1 | 2 | High risk of bias |
| Boyanovsky et al (16) | 1 | 0 | 2 | 3 | High risk of bias |
| Cernera et al (17) | 3 | 0 | 2 | 5 | Intermediate risk of bias |
| Chen et al (18) | 2 | 0 | 1 | 3 | High risk of bias |
| Coen et al (19) | 2 | 0 | 2 | 4 | Intermediate risk of bias |
| Cumming et al (20) | 4 | 1 | 2 | 7 | Low risk of bias |
| Daraban et al (111) | 4 | 0 | 2 | 6 | Intermediate risk of bias |
| De Moerloose (22) | 3 | 0 | 2 | 5 | Intermediate risk of bias |
| de Paula Sabino et al (23) | 2 | 0 | 2 | 4 | Intermediate risk of bias |
| de Visser et al (24) | 1 | 0 | 2 | 3 | High risk of bias |
| Delahousse et al (25) | 3 | 0 | 2 | 5 | Intermediate risk of bias |
| Delluc et al (26) | 3 | 0 | 2 | 5 | Intermediate risk of bias |
| Di Minno et al (27) | 4 | 2 | 2 | 8 | Low risk of bias |
| Dimri et al (28) | 4 | 0 | 2 | 6 | Intermediate risk of bias |
| Djordjevic et al (29) | 2 | 0 | 2 | 4 | Intermediate risk of bias |
| Farajzadeh et al (30) | 2 | 0 | 2 | 4 | Intermediate risk of bias |
| Folsom et al (31) | 1 | 0 | 2 | 3 | High risk of bias |
| Gorski et al (32) | 4 | 1 | 2 | 7 | Low risk of bias |
| Heijboer et al (33) | 4 | 1 | 2 | 7 | Low risk of bias |
| Hillarp et al (34) | 3 | 2 | 2 | 7 | Low risk of bias |
| Jackson et al (35) | 3 | 0 | 2 | 5 | Intermediate risk of bias |
| Jusić-Karić et al (36) | 1 | 0 | 2 | 3 | High risk of bias |
| Karasu et al (37) | 2 | 2 | 2 | 6 | Intermediate risk of bias |
| Kalayci et al (38) | 3 | 0 | 2 | 5 | Intermediate risk of bias |
| Keijzer et al (39) | 1 | 2 | 2 | 5 | Intermediate risk of bias |
| Kupeli et al (40) | 2 | 0 | 1 | 3 | High risk of bias |
| Legnani et al (41) | 3 | 2 | 2 | 7 | Low risk of bias |
| Lichy et al (42) | 2 | 1 | 2 | 5 | Intermediate risk of bias |
| Lijfering et al (112) | 3 | 2 | 0 | 5 | Intermediate risk of bias |
| Lindmarker et al (44) | 2 | 2 | 2 | 6 | Intermediate risk of bias |
| Linna et al (45) | 3 | 0 | 2 | 5 | Intermediate risk of bias |
| Mansilha et al (113) | 2 | 0 | 2 | 4 | Intermediate risk of bias |
| Mansilha et al (114) | 3 | 0 | 2 | 5 | Intermediate risk of bias |
| Manten et al (48) | 0 | 2 | 2 | 4 | Intermediate risk of bias |
| Marcucci et al (49) | 4 | 2 | 2 | 8 | Low risk of bias |
| Marcucci et al (50) | 2 | 2 | 2 | 6 | Intermediate risk of bias |
| Mitsuguro et al (51) | 1 | 0 | 1 | 2 | High risk of bias |
| Nizankowska-Mogilnicka et al (52) | 3 | 1 | 2 | 6 | Intermediate risk of bias |
| Obeid et al (53) | 2 | 2 | 2 | 6 | Intermediate risk of bias |
| Okumus et al (54) | 2 | 2 | 2 | 6 | Intermediate risk of bias |
| Pérez-Ceballos et al (55) | 1 | 0 | 2 | 3 | High risk of bias |
| Pestana et al (56) | 2 | 0 | 2 | 4 | Intermediate risk of bias |
| Primignani et al (57) | 3 | 2 | 2 | 7 | Low risk of bias |
| Rahimi et al (58) | 2 | 0 | 2 | 4 | Intermediate risk of bias |
| Renner et al (59) | 3 | 0 | 2 | 5 | Intermediate risk of bias |
| Ridker et al (60) | 3 | 2 | 2 | 7 | Low risk of bias |
| Rosendaal et al (61) | 3 | 0 | 2 | 5 | Intermediate risk of bias |
| Russo et al (62) | 0 | 0 | 1 | 1 | High risk of bias |
| Sakata et al (115) | 2 | 0 | 2 | 4 | Intermediate risk of bias |
| Salazar-Sanchez et al (116) | 1 | 0 | 2 | 3 | High risk of bias |
| Salomon et al (65) | 3 | 0 | 2 | 5 | Intermediate risk of bias |
| Shen et al (66) | 2 | 0 | 2 | 4 | Intermediate risk of bias |
| Souto et al (67) | 2 | 2 | 2 | 6 | Intermediate risk of bias |
| Svensson et al (68) | 2 | 0 | 2 | 4 | Intermediate risk of bias |
| Tony et al (70) | 3 | 0 | 2 | 5 | Intermediate risk of bias |
| Tosetto et al (69) | 2 | 2 | 1 | 5 | Intermediate risk of bias |
| Trégouët et al (71) | 3 | 0 | 2 | 5 | Intermediate risk of bias |
| Weger et al (72) | 2 | 0 | 2 | 4 | Intermediate risk of bias |
| Weih et al (73) | 3 | 0 | 2 | 5 | Intermediate risk of bias |
| Zalavras Ch et al (74) | 3 | 0 | 2 | 5 | Intermediate risk of bias |
| Zerjavic et al (75) | 0 | 0 | 2 | 2 | High risk of bias |
| Zhang et al (117) | 2 | 1 | 2 | 5 | Intermediate risk of bias |

| Newcastle Ottawa Score for included cohort studies Median score = 7 | | | | | |
| --- | --- | --- | --- | --- | --- |
| **Study** | **Selection** (max. four stars) | **Comparability** (max. two stars) | **Outcome** (max. three stars) | **Overall score** (max. 9 stars) | **Evaluation** |
| Brouwer et al (77) | 2 | 0 | 2 | 4 | Intermediate risk of bias |
| Castaman et al (78) | 3 | 0 | 0 | 3 | High risk of bias |
| Cohen et al (79) | 4 | 0 | 1 | 5 | Intermediate risk of bias |
| Coppens et al (80) | 4 | 0 | 2 | 6 | Intermediate risk of bias |
| Couturaud et al (118) | 4 | 0 | 3 | 7 | Low risk of bias |
| De Stefano et al (82) | 3 | 1 | 2 | 6 | Intermediate risk of bias |
| De Stefano et al (83) | 4 | 0 | 2 | 6 | Intermediate risk of bias |
| Di Minno et al (84) | 4 | 0 | 3 | 7 | Low risk of bias |
| Eichinger et al (85) | 4 | 1 | 2 | 7 | Low risk of bias |
| Evensen et al (119) | 4 | 1 | 2 | 7 | Low risk of bias |
| Hodeib et al (120) | 4 | 1 | 3 | 8 | Low risk of bias |
| Lijfering et al (88) | 4 | 2 | 3 | 9 | Low risk of bias |
| Mahmoodi et al (89) | 4 | 2 | 2 | 8 | Low risk of bias |
| Makris et al (90) | 3 | 2 | 2 | 7 | Low risk of bias |
| Manderstedt et al (121) | 4 | 2 | 2 | 8 | Low risk of bias |
| Mateo et al (92) | 3 | 2 | 1 | 6 | Intermediate risk of bias |
| Méan et al (93) | 4 | 2 | 3 | 9 | Low risk of bias |
| Middeldorp et al (94) | 4 | 1 | 2 | 7 | Low risk of bias |
| Miles et al (95) | 2 | 2 | 2 | 6 | Intermediate risk of bias |
| Olivo et al (96) | 4 | 2 | 2 | 8 | Low risk of bias |
| Pires et al (97) | 2 | 0 | 2 | 4 | Intermediate risk of bias |
| Puhr et al (122) | 4 | 2 | 2 | 8 | Low risk of bias |
| Ridker et al (99) | 3 | 2 | 3 | 8 | Low risk of bias |
| Satpanich et al (100) | 4 | 1 | 2 | 7 | Low risk of bias |
| Simioni et al (101) | 4 | 0 | 2 | 6 | Intermediate risk of bias |
| Simioni et al (102) | 4 | 0 | 3 | 7 | Low risk of bias |
| Tirado et al (103) | 4 | 2 | 2 | 8 | Low risk of bias |
| Tormene et al (104) | 2 | 0 | 2 | 4 | Intermediate risk of bias |
| van Boven et al (105) | 3 | 2 | 0 | 5 | Intermediate risk of bias |
| Weingarz et al (106) | 4 | 2 | 2 | 8 | Low risk of bias |
| Zöller et al (123) | 4 | 2 | 3 | 9 | Low risk of bias |

## Forest plots

### FVL heterozygous Forest Plot


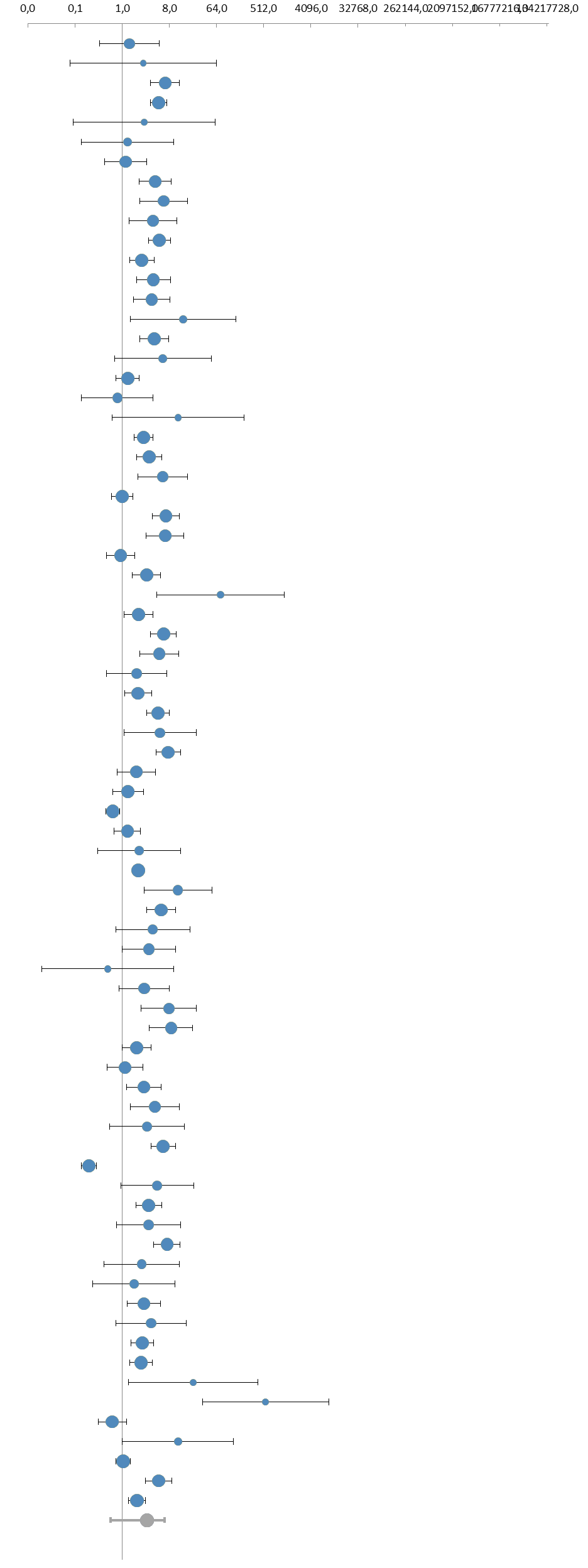


FVL heterozygous Forest Plot, primary VTE


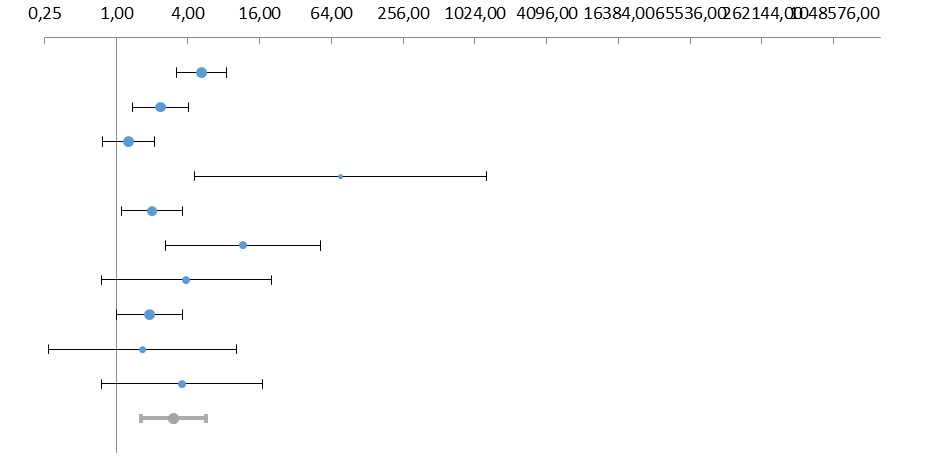


FVL heterozygous Forest Plot, recurrent VTE


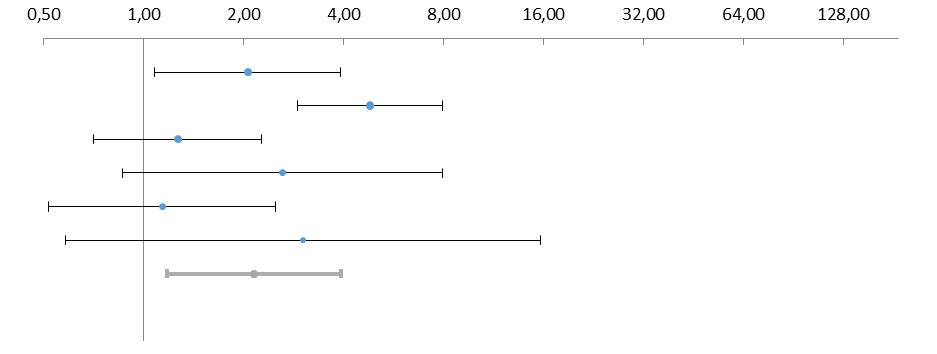


FVL heterozygous Forest Plot, High Quality studies


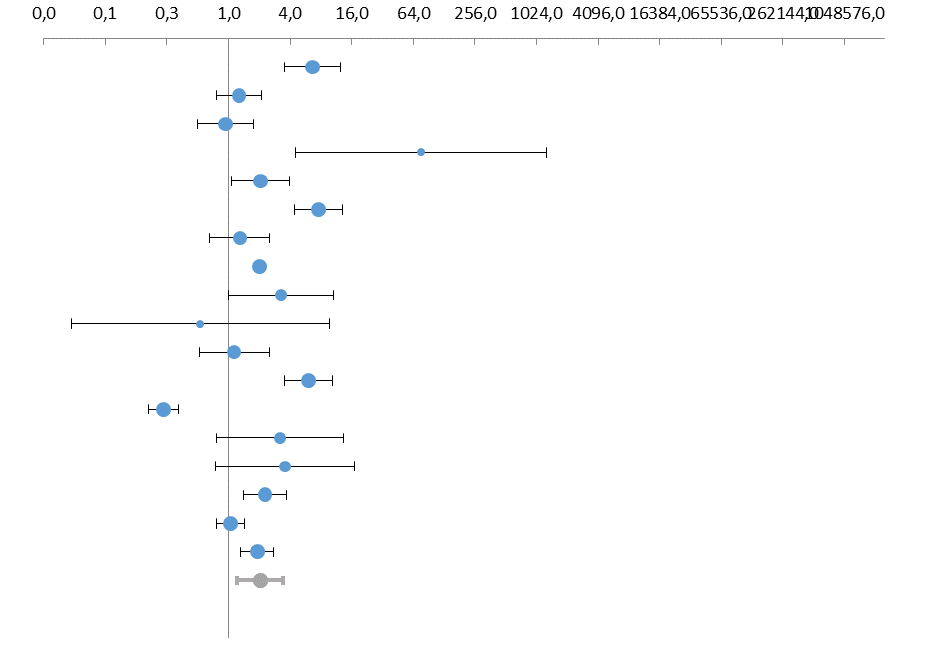


FVL heterozygous Forest plot, Case-control studies


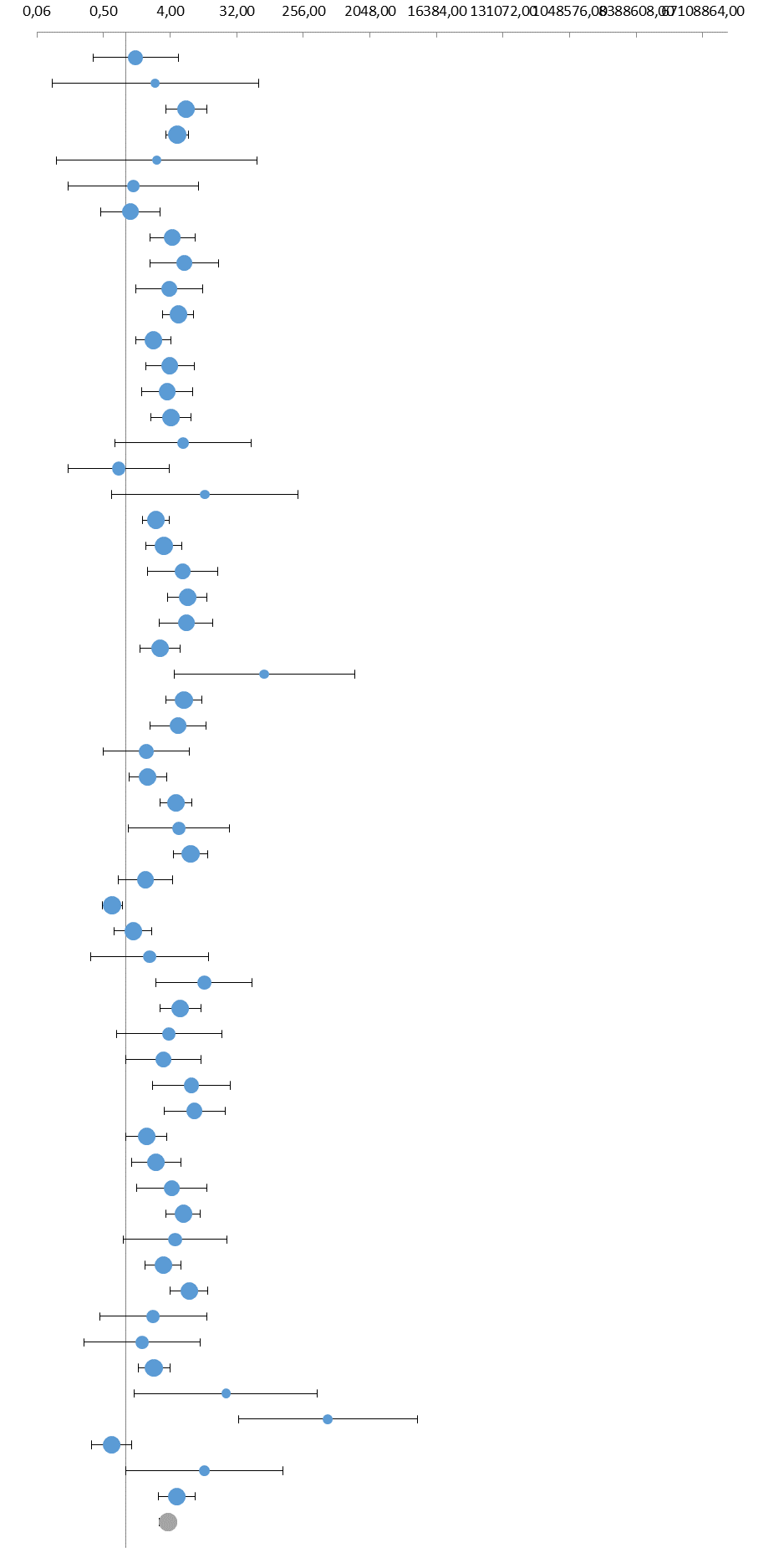


FVL heterozygous Forest Plot, Cohort studies


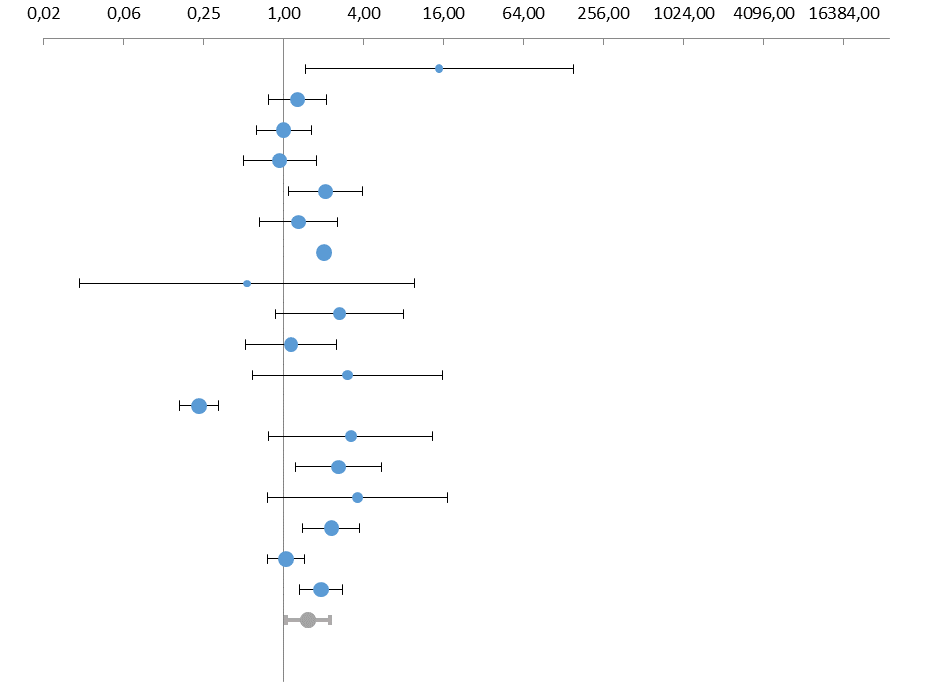


### FVL homozygous Forest Plot


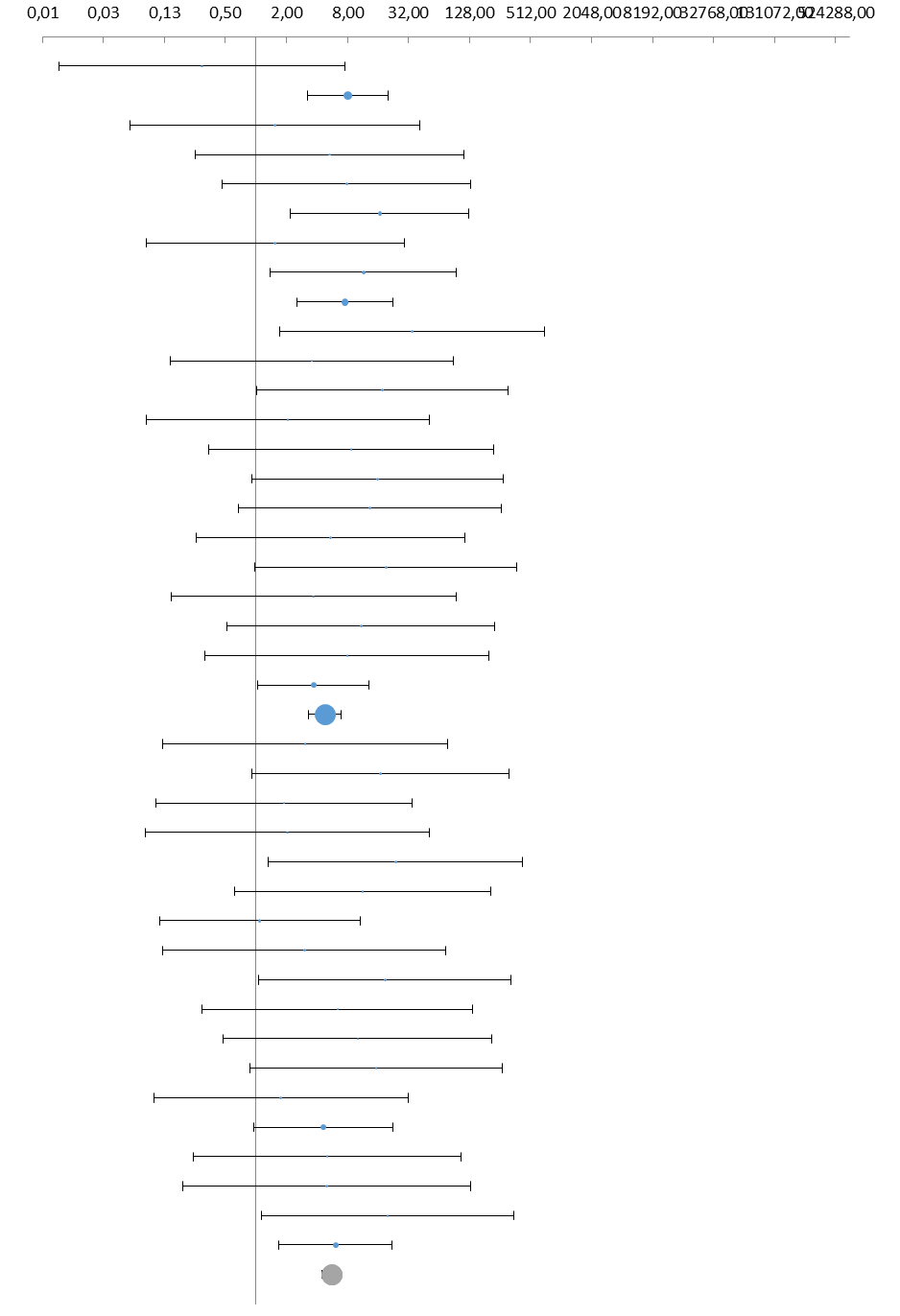


FVL homozygous Forest Plot, primary VTE


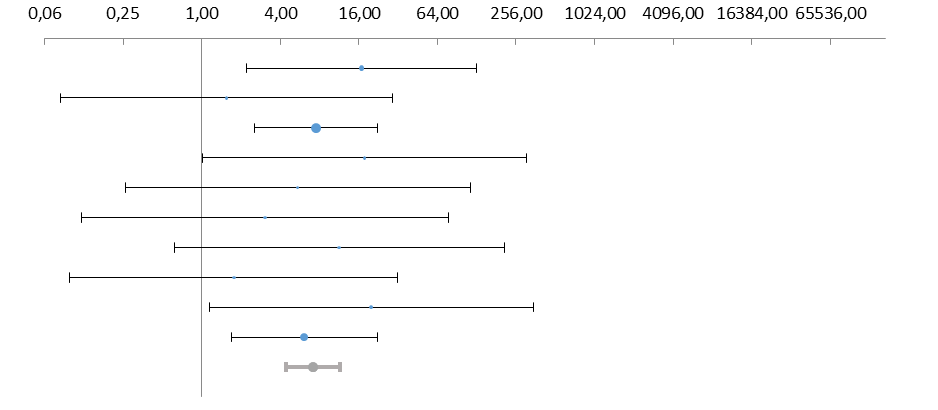


FVL homozygous Forest plot, recurrent VTE


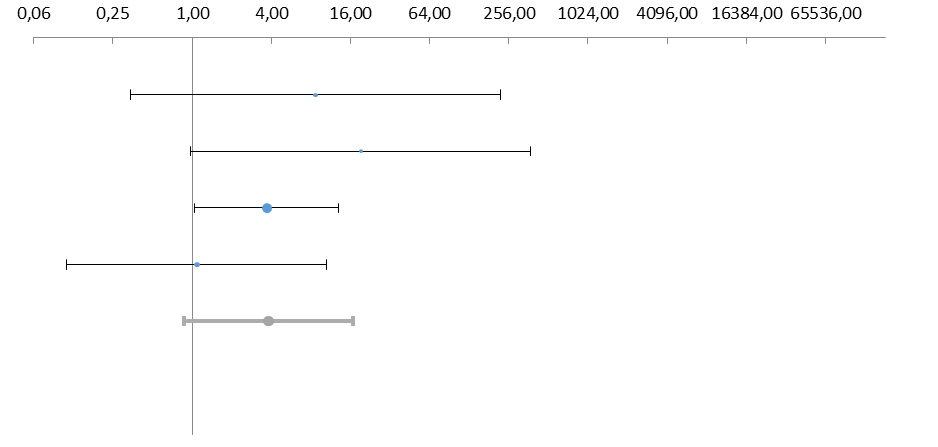


FVL homozygous Forest Plot, High Quality studies


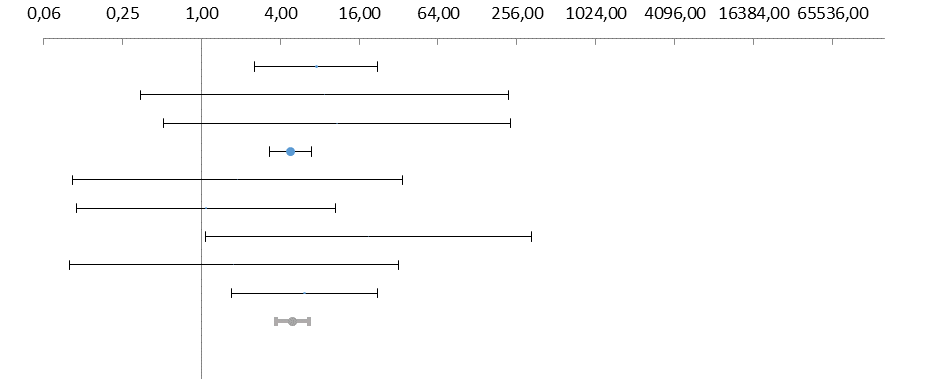


### Prothrombin G20210A heterozygous Forest Plot


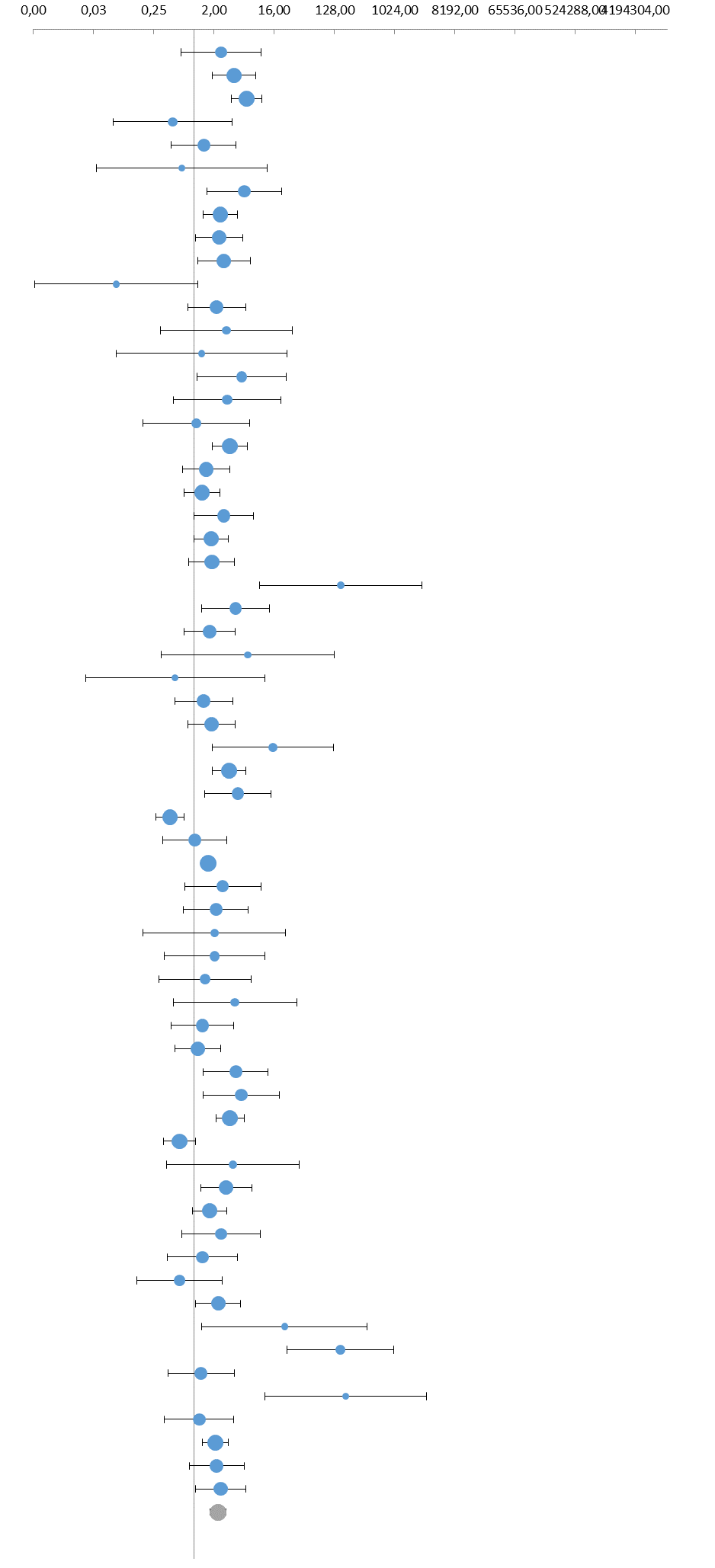


Prothrombin G20210A heterozygous Forest plot, primary VTE


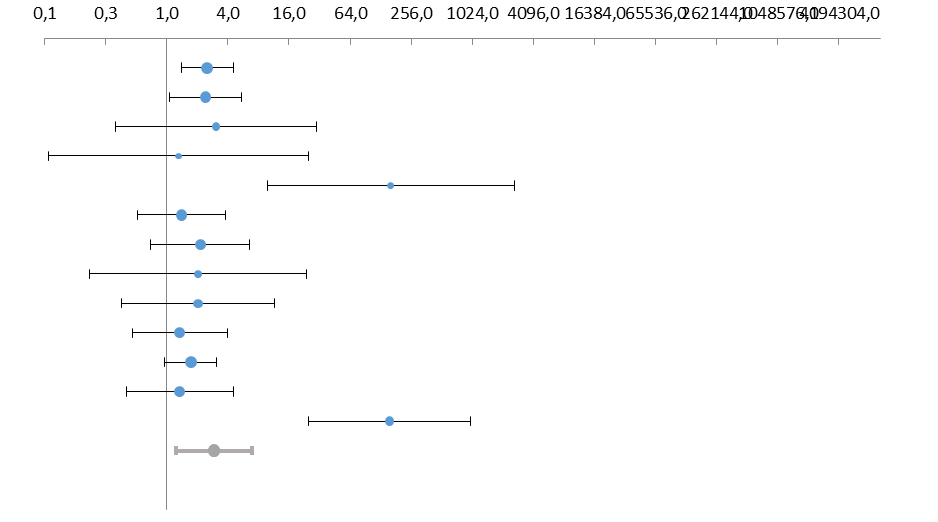


Prothrombin G20210A heterozygous Forest plot, recurrent VTE


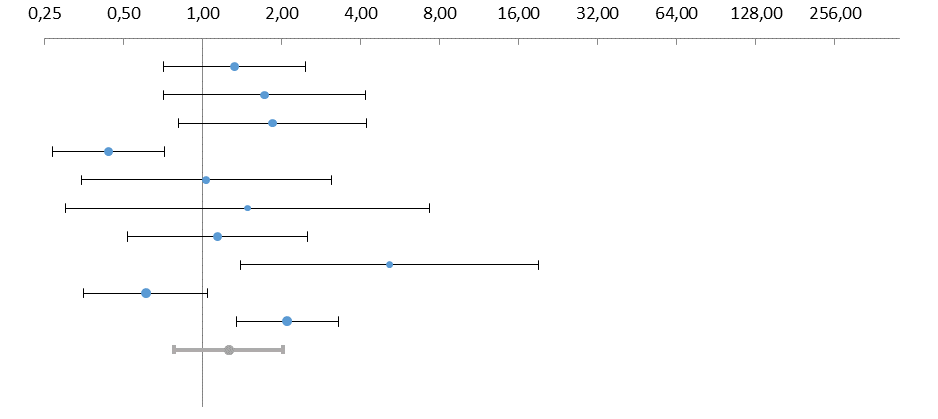


Prothrombin G20210A heterozygous Forest plot, High Quality studies


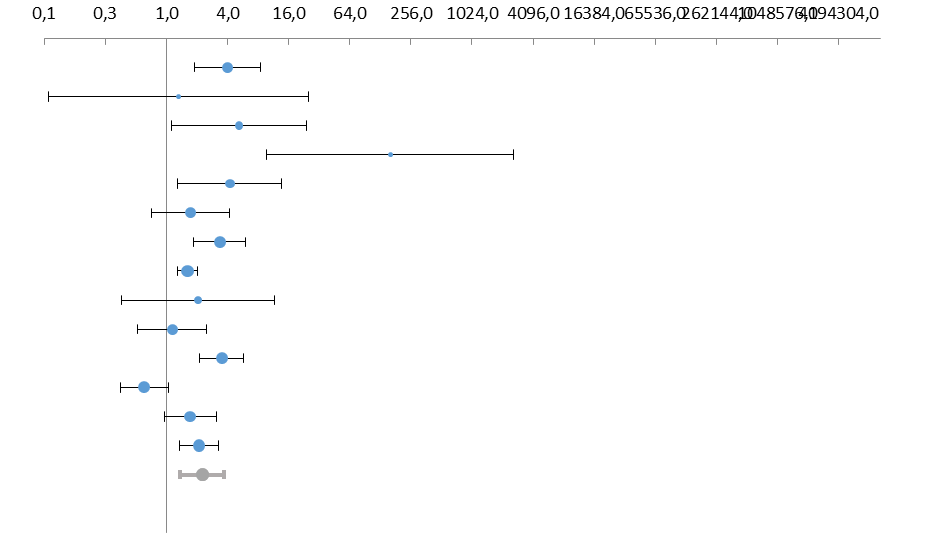


Prothrombin G20210A heterozygous Forest plot, Case-control studies


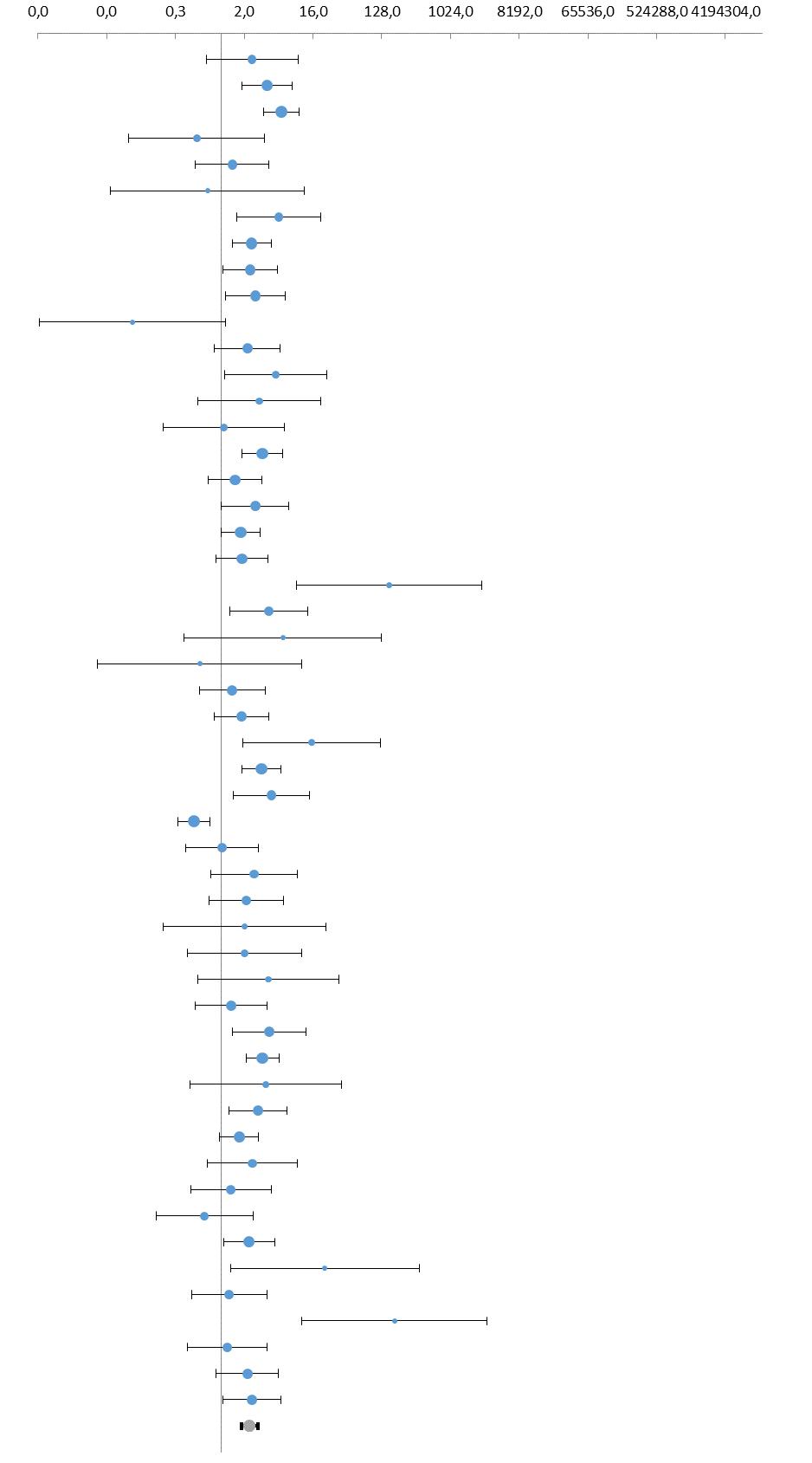


Prothrombin G20210A heterozygous Forest plot, Cohort studies


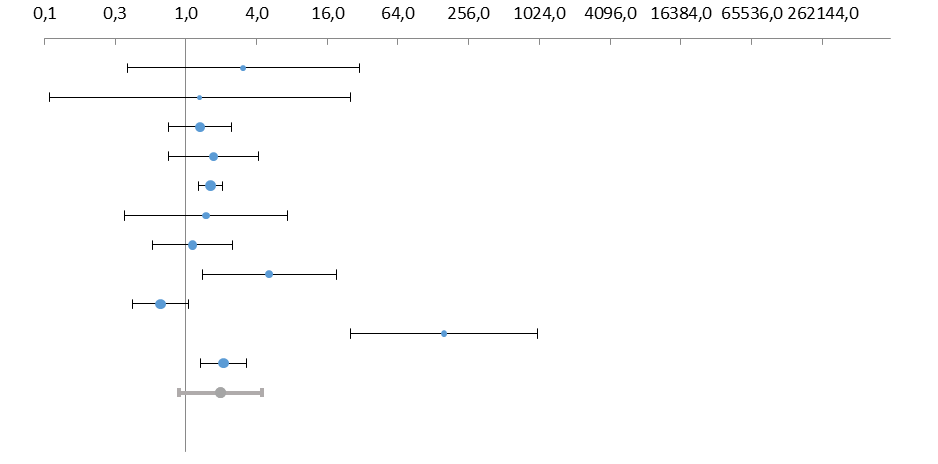


### Prothrombin G20210A homozygous Forest Plot


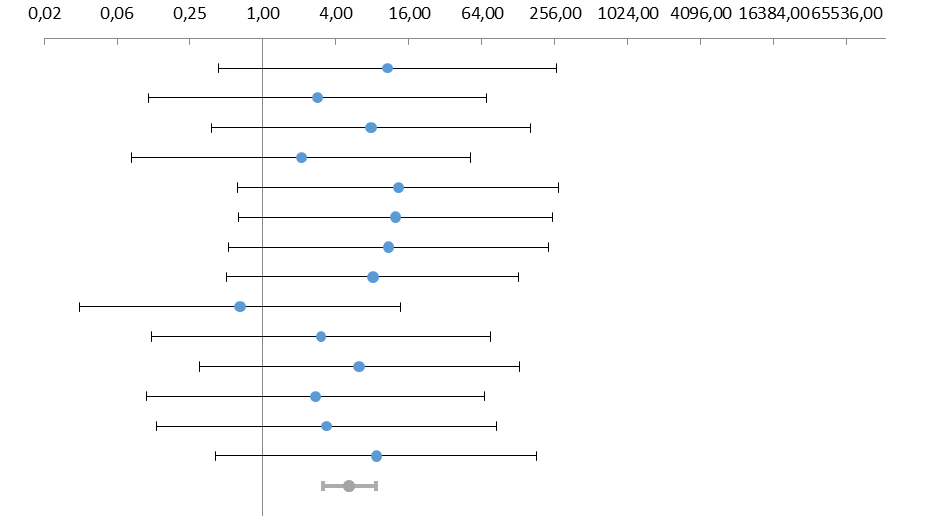


Prothrombin G20210A homozygous Forest plot, primary VTE


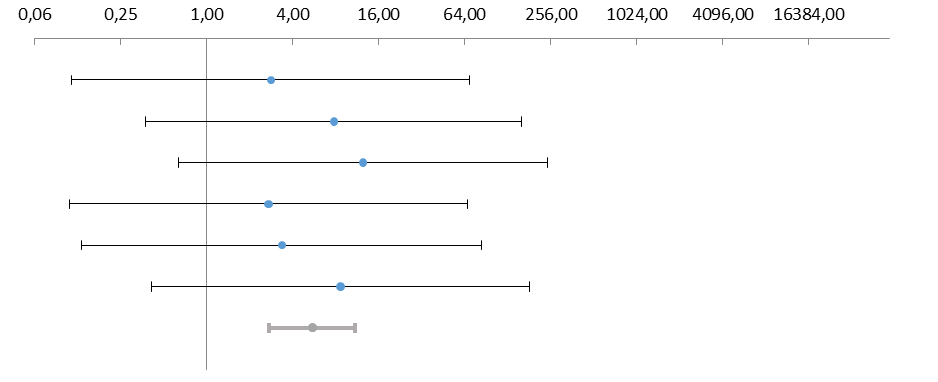


Prothrombin G20210A homozygous, Forest plot, High Quality Studies


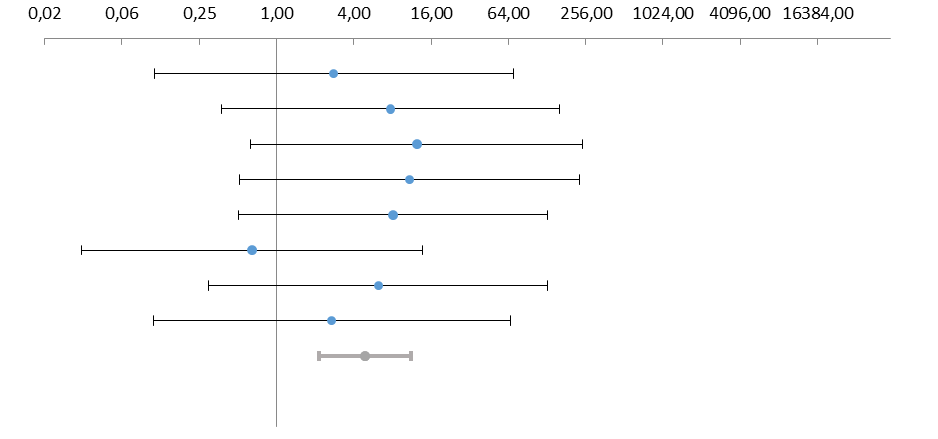


### Compound heterozygous Factor V Leiden and prothrombin G20210A Forest Plot


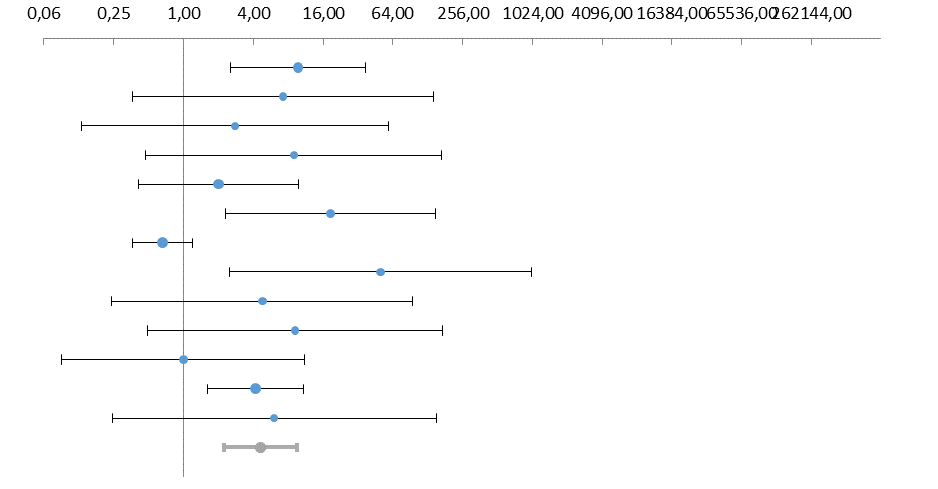


Compound heterozygous Factor V Leiden and prothrombin G20210A Forest plot, primary VTE


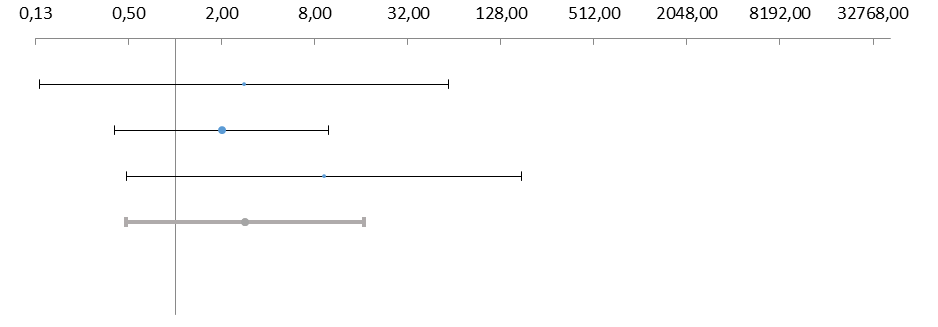


Compound heterozygous Factor V Leiden and prothrombin G20210A Forest plot, High Quality studies


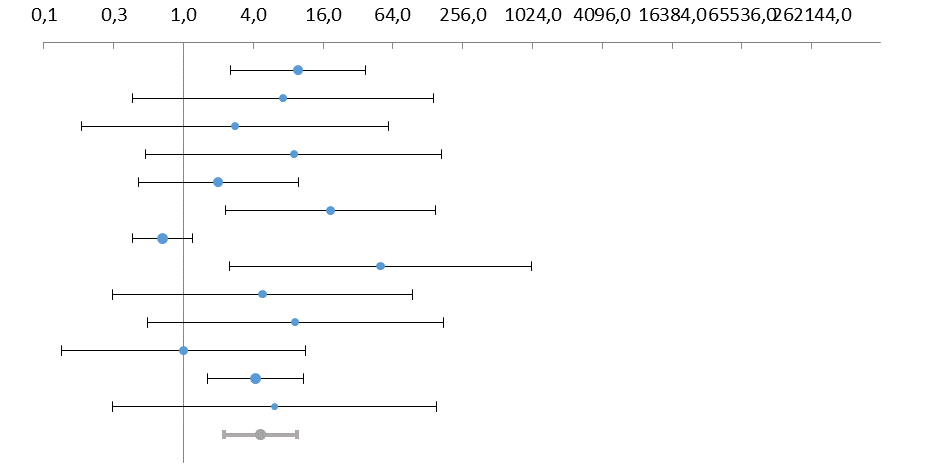


Compound heterozygous Factor V Leiden and prothrombin G20210A Forest plot, Case-control studies


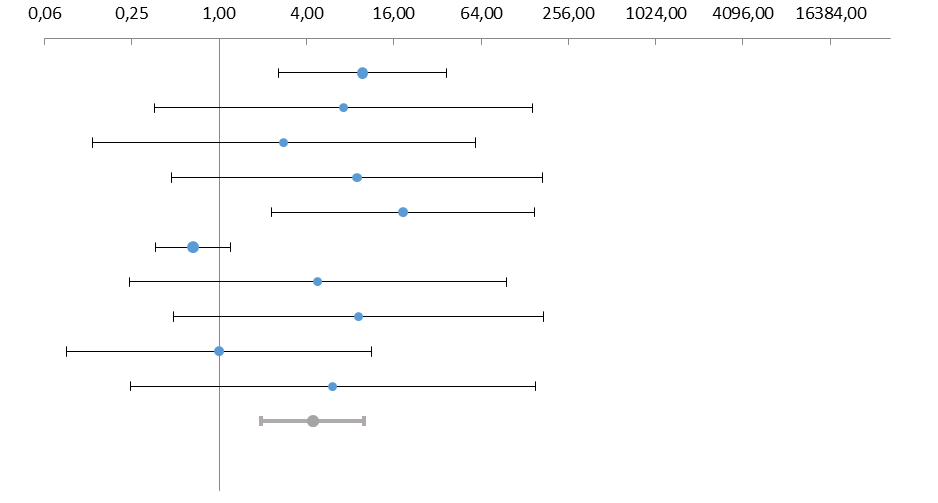


Compound heterozygous Factor V Leiden and prothrombin G20210A Forest plot, Cohort studies


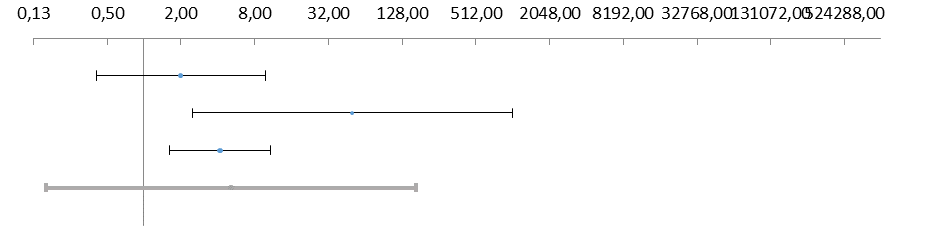


### PC deficiency Forest Plot


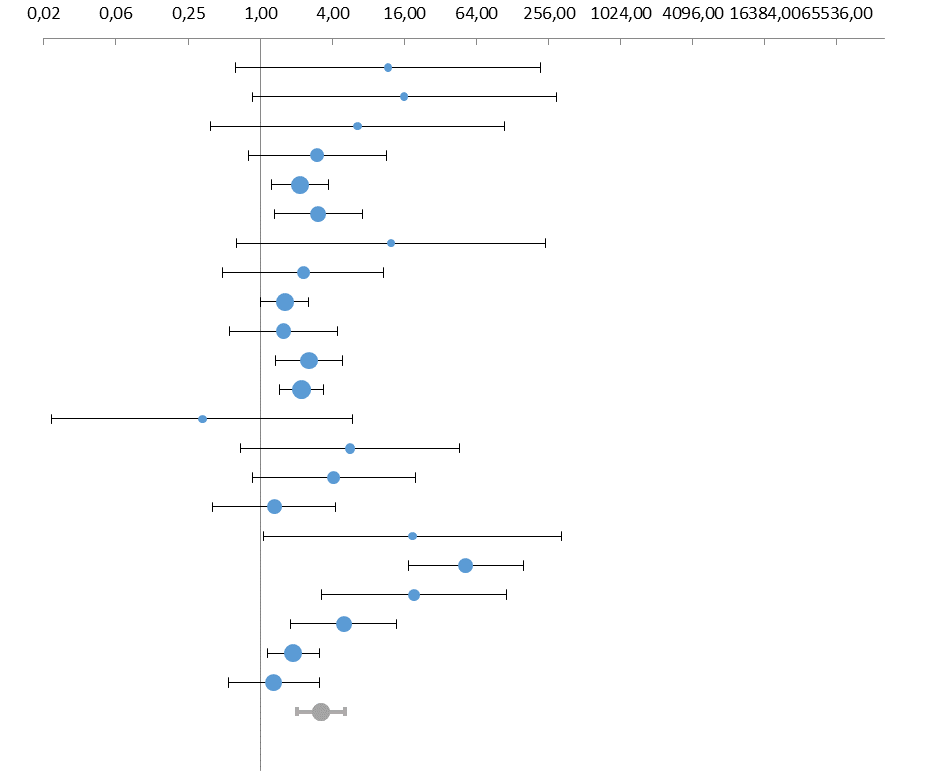


PC deficiency Forest plot, primary VTE


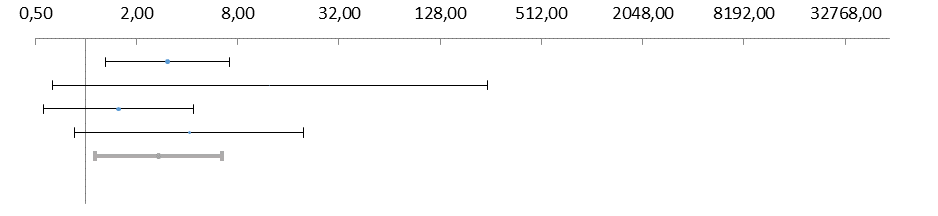


PC deficiency Forest plot, recurrent VTE


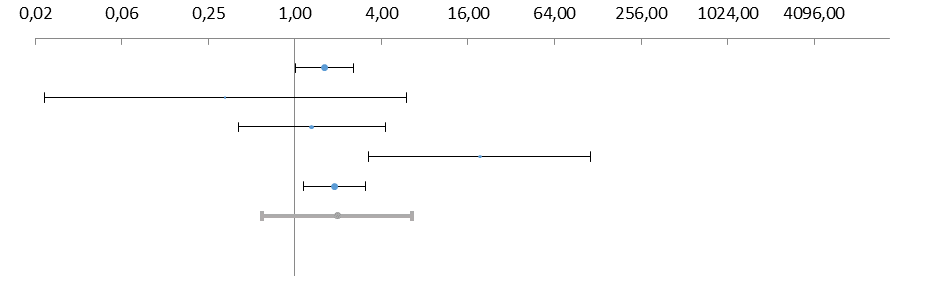


PC deficiency Forest plot, High Quality studies


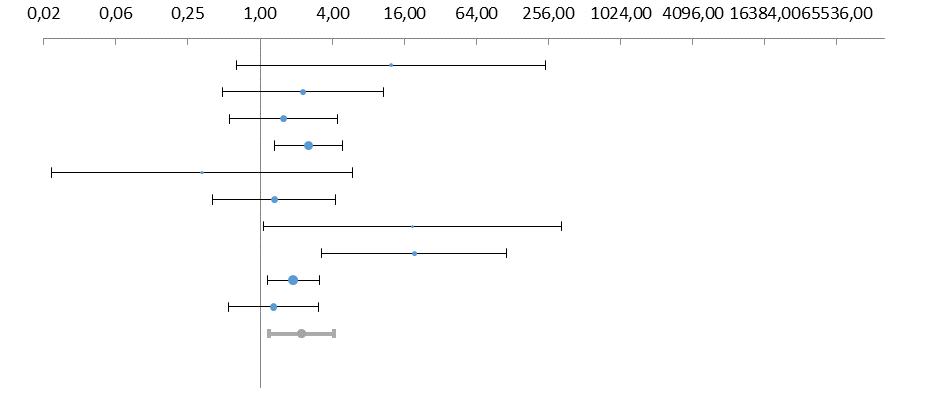


PC deficiency Forest plot, Case-control studies


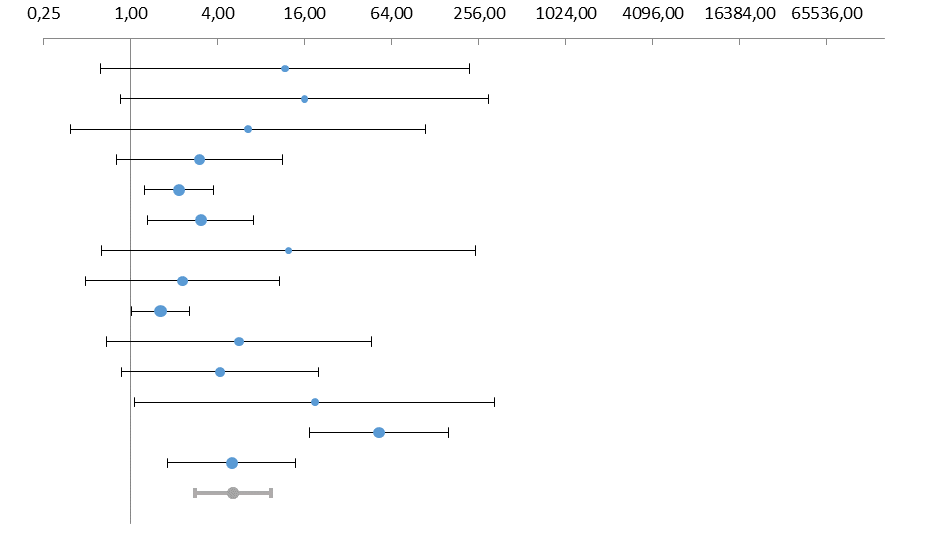


PC deficiency Forest plot, Cohort studies


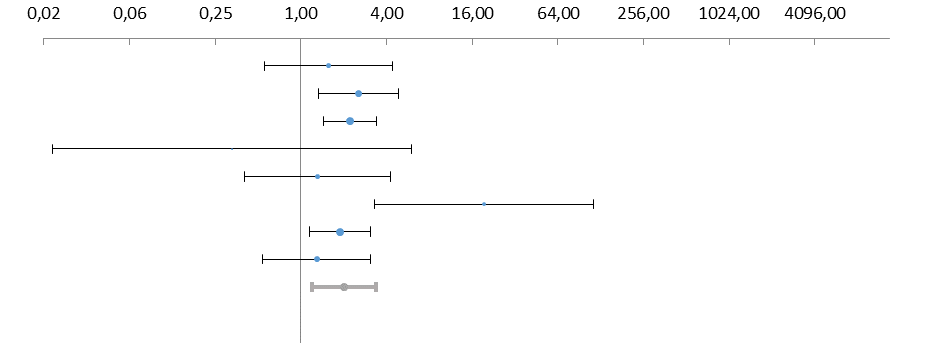


### PS deficiency Forest Plot


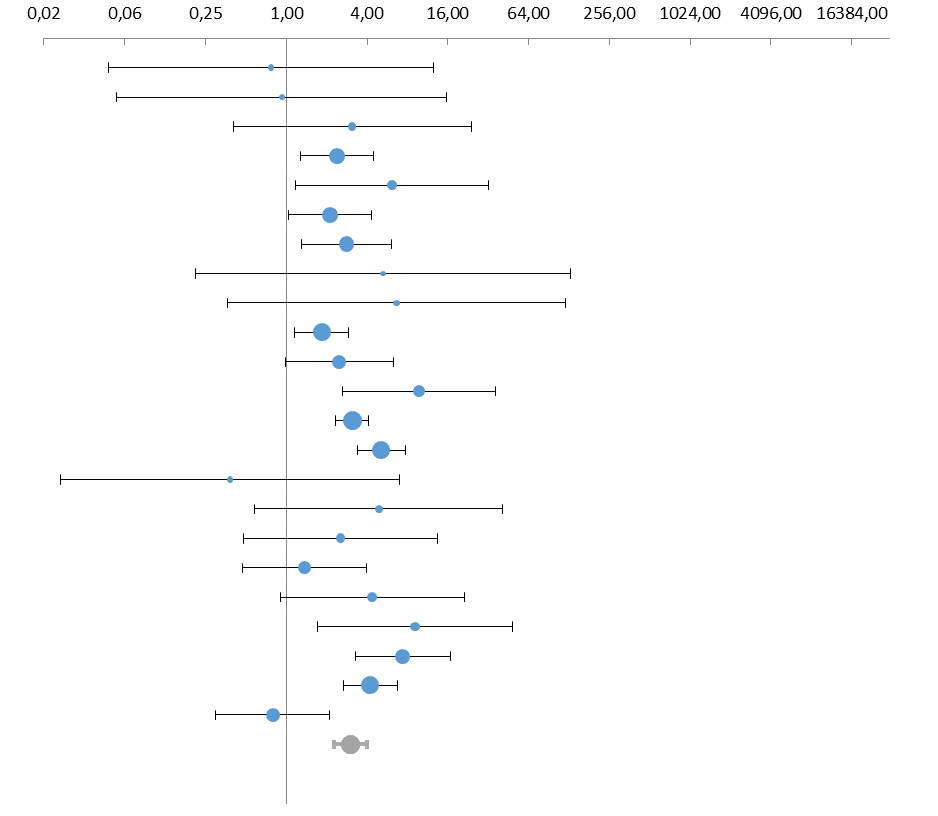


PS deficiency Forest plot, primary VTE


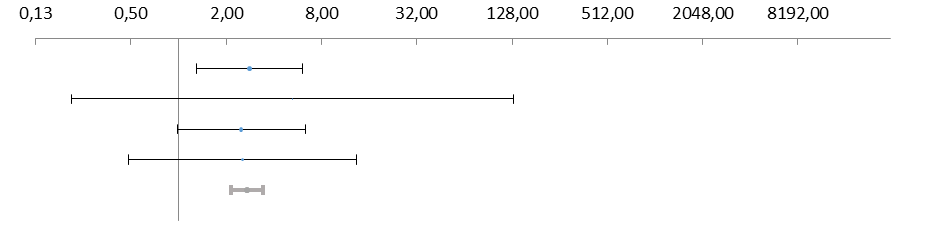


PS deficiency Forest plot, recurrent VTE


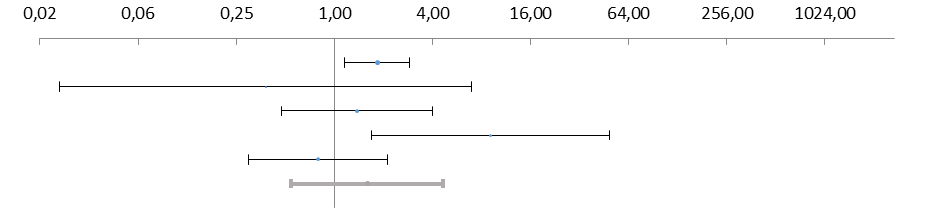


PS deficiency Forest plot, High Quality studies


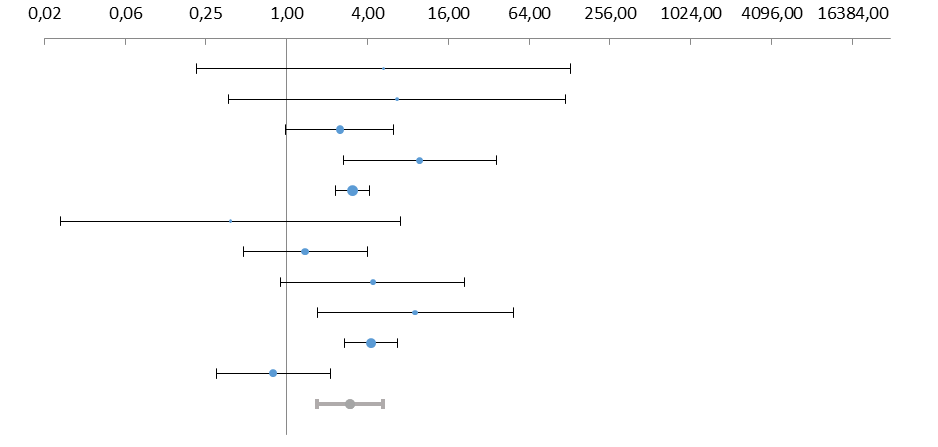


### AT deficiency Forest Plot


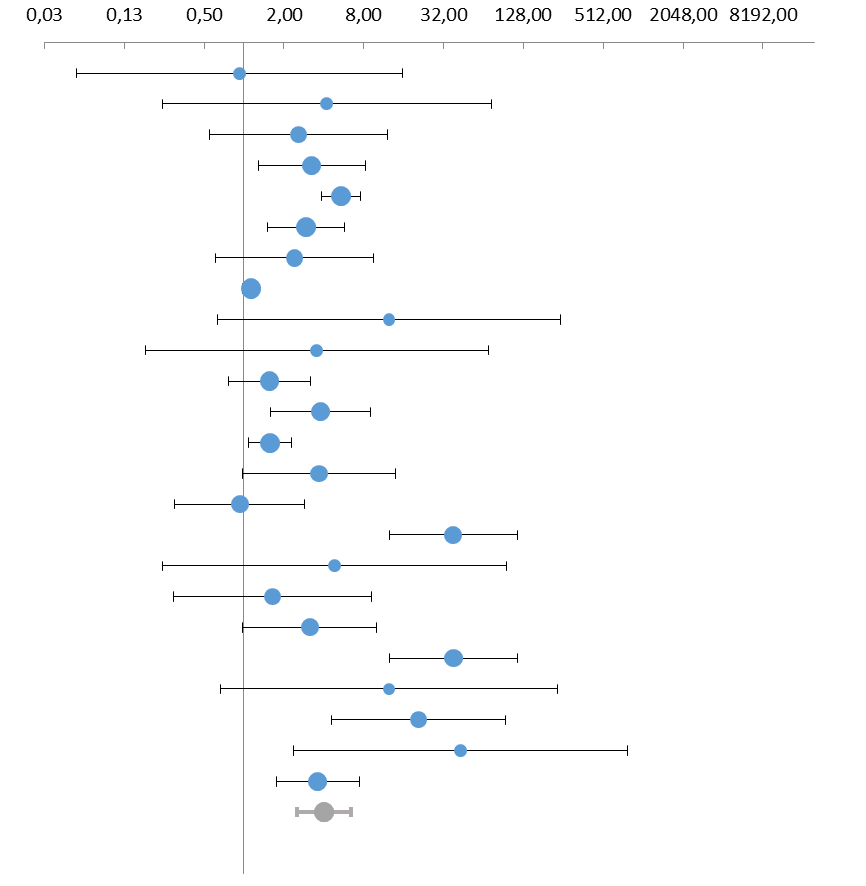


AT deficiency Forest plot, primary VTE


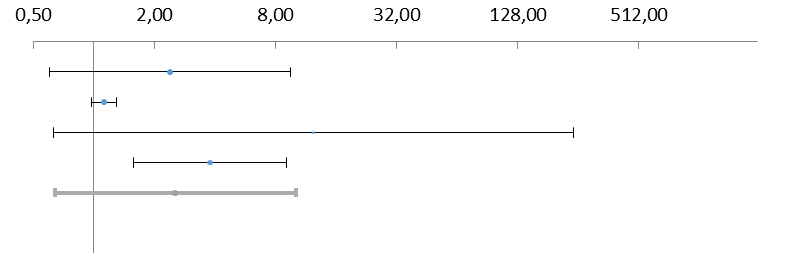


AT deficiency Forest plot, recurrent VTE


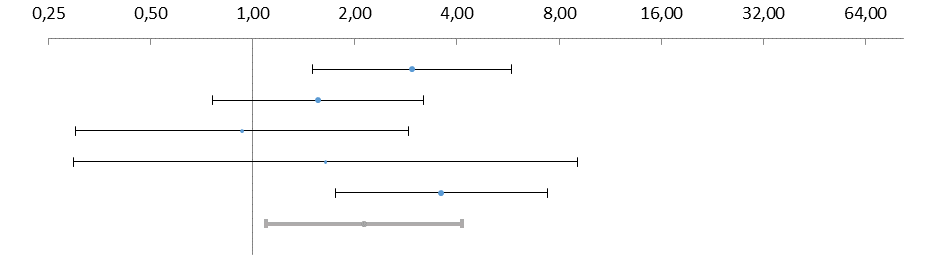


AT deficiency Forest plot, High Quality studies


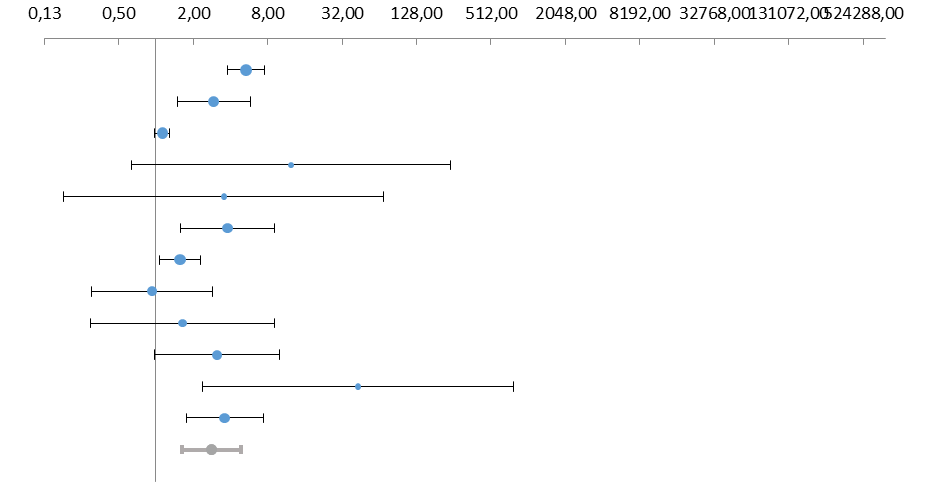


AT deficiency Forest plot, Case-control studies


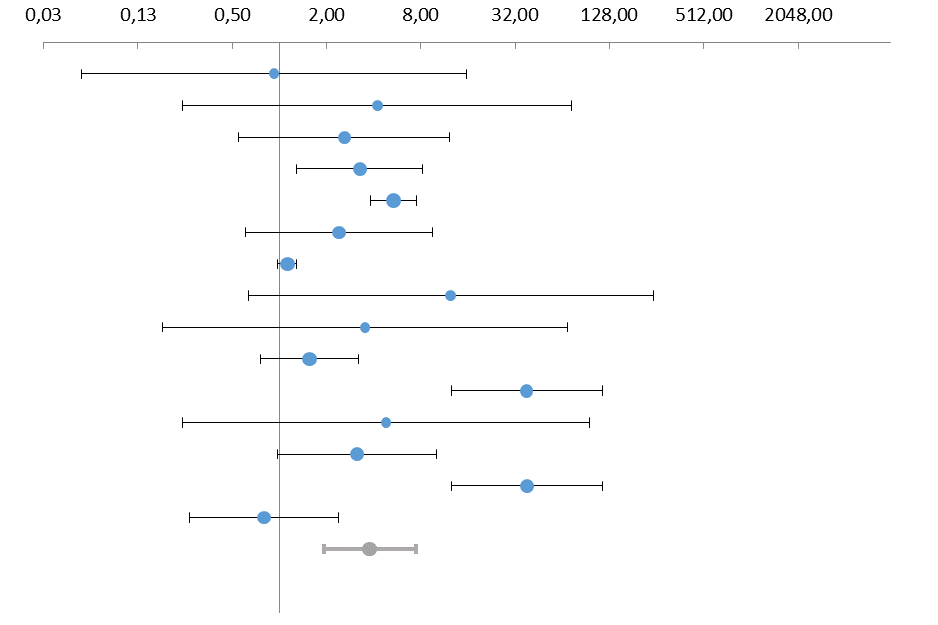


AT deficiency Forest plot, Cohort studies


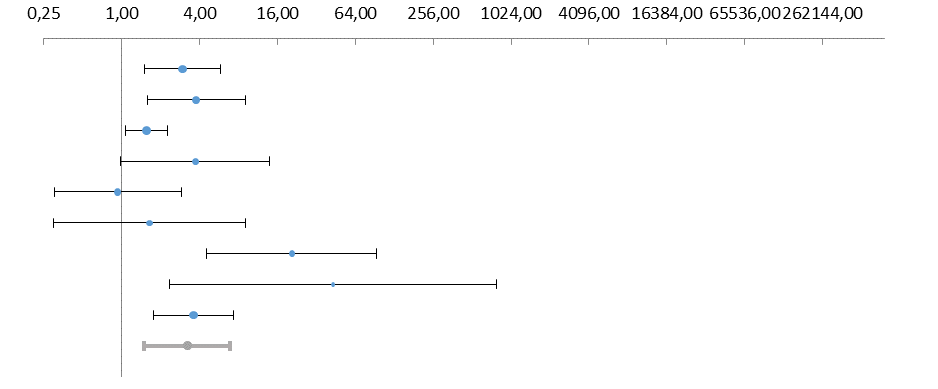


## Publication Bias

## Egger’s Regression Test

| ***Egger’s regression intercept (95% CI)*** | | | | | | | |
| --- | --- | --- | --- | --- | --- | --- | --- |
| Thrombophilia | **Overall** | **Primary VTE** | **Recurrent VTE** |  | **High Quality studies** | **Case-control studies** | **Cohort studies** |
| **Factor V Leiden heterozygous** | 1.72 (0.59 - 2.84)* | 2.05 (-0.27 - 4.37) | \ |  | 2.00 (-1.27 - 5.27) | 1.29 (0.16 - 2.42)* | 1.82 (-0.63 - 4.28) |
| **Factor V Leiden homozygous** | 0.17 (-0.18 - 0.51) | -0.19 (-1.41 - 1.03) | \ |  | \ | NA | NA |
| **Prothrombin G20210A heterozygous** | 1.05 (-0.06 - 2.15) | 1.36 (-0.68 - 3.41) | 2.98 (-1.66 - 7.63) |  | 1.91 (-0.36 - 4.19) | 0.79 (-0.47- 2.05) | 1.98 (-0.89 - 4.84) |
| **Prothrombin G20210A homozygous** | -5.12 (-12.45 - 2.21) | \ | \ |  | \ | NA | NA |
| **Compound heterozygous Factor V Leiden and prothrombin G20210A** | 1.68 (-0.20 - 3.57) | \ | \ |  | 1.68 (-0.20 - 3.57) | 1.65 (-0.77 - 4.06) | \ |
| **Protein C deficiency** | 1.34 (0.15 - 2.53)* | \ | \ |  | 0.78 (-0.93 - 2.50) | 1.78 (0.27 - 3.29)* | \ |
| **Protein S deficiency** | -0.15 (-1.22 - 0.92) | \ | \ |  | 0.12 (-2.01 - 2.24) | NA | NA |
| **Antithrombin deficiency** | 1.10 (-0.57 - 2.77) | \ | \ |  | 1.42 (-0.47 - 3.31) | 0.42 (-2.14 - 2.98) | \ |

NA: subgroup analysis not warranted, \: insufficient data, * : *p* < 0.05 indicating funnel plot asymmetry

### FVL heterozygous Funnel Plot

FVL heterozygous Funnel plot, primary VTE

FVL heterozygous Funnel plot, High Quality studies

FVL heterozygous Funnel plot, Case-control studies

FVL heterozygous Funnel plot, Cohort studies

### FVL homozygous Funnel Plot

FVL homozygous Funnel plot, primary VTE

### Prothrombin G20210A heterozygous Funnel Plot

Prothrombin G20210A heterozygous Funnel plot, primary VTE

Prothrombin G20210A heterozygous Funnel plot, recurrent VTE

Prothrombin G20210A heterozygous Funnel plot, High Quality studies

Prothrombin G20210A heterozygous Funnel plot, Case-control studies

Prothrombin G20210A heterozygous Funnel plot, Cohort studies

### Prothrombin G20210A homozygous Funnel Plot

### Compound FVL and FII heterozygous Funnel Plot

Compound FVL and FII heterozygous Funnel plot, High Quality studies

Compound FVL and FII heterozygous Funnel plot, Case-control studies

### PC deficiency Funnel Plot

PC deficiency Funnel plot, High Quality studies

PC deficiency Funnel plot, Case-control studies

### PS deficiency Funnel Plot

PS deficiency Funnel plot, High Quality studies

### AT deficiency Funnel Plot

AT deficiency Funnel plot, High Quality studies

AT deficiency Funnel plot, Case-control studies

## References

1. Aleksova A, Di Nucci M, Gobbo M, et al. Factor-V HR2 haplotype and thromboembolic disease. Acta Cardiol. 2015;70(6):707-11 <http://dx.doi.org/10.2143/AC.70.6.3120184>.

2. Alfeel A, Abdalhabib E, Mobarki A, Hamali H, ibrahim I, Elzaki S. Frequency of Factor V Leiden and Prothrombin G20210A Mutations in Sudanese Patients with Deep Vein Thrombosis in Khartoum Hospitals. Pakistan Journal of Medical and Health Sciences. 2020

3. Alhenc-Gelas M, Arnaud E, Nicaud V, et al. Venous thromboembolic disease and the prothrombin, methylene tetrahydrofolate reductase and factor V genes. Thromb Haemost. 1999;81(4):506-10

4. Almawi WY, Tamim H, Kreidy R, et al. A case control study on the contribution of factor V-Leiden, prothrombin G20210A, and MTHFR C677T mutations to the genetic susceptibility of deep venous thrombosis. J Thromb Thrombolysis. 2005;19(3):189-96 <http://dx.doi.org/10.1007/s11239-005-1313-x>.

5. Altinisik J, Ates O, Ulutin T, Cengiz M, Buyru N. Factor V Leiden, prothrombin G20210A, and protein C mutation frequency in Turkish venous thrombosis patients. Clin Appl Thromb Hemost. 2008;14(4):415-20 <http://dx.doi.org/10.1177/1076029607306404>.

6. Aras S, Yilmaz G, Alpas I, Baltaci V, Tayanc E, Aydin P. Retinal vein occlusion and factor V Leiden and prothrombin 20210 G:A mutations. Eur J Ophthalmol. 2001;11(4):351-5 <http://dx.doi.org/10.1177/112067210101100406>.

7. Arsene S, Delahousse B, Regina S, Le Lez ML, Pisella PJ, Gruel Y. Increased prevalence of factor V Leiden in patients with retinal vein occlusion and under 60 years of age. Thromb Haemost. 2005;94(1):101-6 <http://dx.doi.org/10.1160/TH04-10-0659>.

8. Arsov T, Miladinova D, Spiroski M. Factor V Leiden is associated with higher risk of deep venous thrombosis of large blood vessels. Croat Med J. 2006;47(3):433-9

9. Ates O. The deficiencies of protein C, protein S and antithrombin III in patients with retinal vein occlusion: a Turkish sample. Clin Lab Haematol. 2006;28(6):391-2 <http://dx.doi.org/10.1111/j.1365-2257.2006.00834.x>.

10. Ben Salem-Berrabah O, Fekih-Mrissa N, N'Siri B, et al. Thrombophilic polymorphisms - factor V Leiden G1691A, prothrombin G20210A and MTHFR C677T - in Tunisian patients with cerebral venous thrombosis. J Clin Neurosci. 2012;19(9):1326-7 <http://dx.doi.org/10.1016/j.jocn.2011.11.029>.

11. Beye A, Pindur G. Clinical significance of factor V Leiden and prothrombin G20210A-mutations in cerebral venous thrombosis - comparison with arterial ischemic stroke. Clin Hemorheol Microcirc. 2017;67(3-4):261-6 <http://dx.doi.org/10.3233/CH-179207>.

12. Bezgin T, Kaymaz C, Akbal O, Yilmaz F, Tokgoz HC, Ozdemir N. Thrombophilic Gene Mutations in Relation to Different Manifestations of Venous Thromboembolism: A Single Tertiary Center Study. Clin Appl Thromb Hemost. 2018;24(1):100-6 <http://dx.doi.org/10.1177/1076029616672585>.

13. Blom JW, Doggen CJ, Osanto S, Rosendaal FR. Old and new risk factors for upper extremity deep venous thrombosis. J Thromb Haemost. 2005;3(11):2471-8 <http://dx.doi.org/10.1111/j.1538-7836.2005.01625.x>.

14. Bombeli T, Basic A, Fehr J. Prevalence of hereditary thrombophilia in patients with thrombosis in different venous systems. Am J Hematol. 2002;70(2):126-32 <http://dx.doi.org/10.1002/ajh.10103>.

15. Bouaziz-Borgi L, Nguyen P, Hezard N, Musharrafieh U, Almawi WY, Mahjoub T. A case control study of deep venous thrombosis in relation to factor V G1691A (Leiden) and A4070G (HR2 Haplotype) polymorphisms. Exp Mol Pathol. 2007;83(3):480-3 <http://dx.doi.org/10.1016/j.yexmp.2007.04.006>.

16. Boyanovsky B, Russeva M, Ganev V, Penev M, Baleva M. Prevalence of factor V Leiden and prothrombin 20210 A variant in Bulgarian patients with pulmonary thromboembolism and deep venous thrombosis. Blood Coagul Fibrinolysis. 2001;12(8):639-42 <http://dx.doi.org/10.1097/00001721-200112000-00004>.

17. Cernera G, Di Minno A, Amato F, et al. Molecular Analysis of Prothrombotic Gene Variants in Venous Thrombosis: A Potential Role for Sex and Thrombotic Localization. J Clin Med. 2020;9(4) <http://dx.doi.org/10.3390/jcm9041008>.

18. Chen TY, Su WC, Tsao CJ. Incidence of thrombophilia detected in southern Taiwanese patients with venous thrombosis. Ann Hematol. 2003;82(2):114-7 <http://dx.doi.org/10.1007/s00277-002-0603-z>.

19. Coen D, Zadro R, Honovic L, Banfic L, Stavljenic Rukavina A. Prevalence and association of the factor V Leiden and prothrombin G20210A in healthy subjects and patients with venous thromboembolism. Croat Med J. 2001;42(4):488-92

20. Cumming AM, Keeney S, Salden A, Bhavnani M, Shwe KH, Hay CR. The prothrombin gene G20210A variant: prevalence in a U.K. anticoagulant clinic population. Br J Haematol. 1997;98(2):353-5 <http://dx.doi.org/10.1046/j.1365-2141.1997.2353052.x>.

21. Daraban A, Trifa A, Popp R-A, et al. Thrombophilia genetic testing in Romanian young women with acute thrombotic events: Role of Factor V Leiden, Prothrombin G20210A, MTHFR C677T and A1298C polymorphisms. Romanian Journal of Laboratory Medicine. 2016;24:291-305 <http://dx.doi.org/10.1515/rrlm-2016-0032>.

22. de Moerloose P, Reber G, Perrier A, Perneger T, Bounameaux H. Prevalence of factor V Leiden and prothrombin G20210A mutations in unselected patients with venous thromboembolism. Br J Haematol. 2000;110(1):125-9 <http://dx.doi.org/10.1046/j.1365-2141.2000.02039.x>.

23. de Paula Sabino A, Guimaraes DA, Ribeiro DD, et al. Increased Factor V Leiden frequency is associated with venous thrombotic events among young Brazilian patients. J Thromb Thrombolysis. 2007;24(3):261-6 <http://dx.doi.org/10.1007/s11239-007-0024-x>.

24. de Visser MC, Guasch JF, Kamphuisen PW, Vos HL, Rosendaal FR, Bertina RM. The HR2 haplotype of factor V: effects on factor V levels, normalized activated protein C sensitivity ratios and the risk of venous thrombosis. Thromb Haemost. 2000;83(4):577-82

25. Delahousse B, Arsene S, Piquemal R, et al. The 20210A allele of the prothrombin gene is not a risk factor for retinal vein occlusion. Blood Coagul Fibrinolysis. 1998;9(5):447-8 <http://dx.doi.org/10.1097/00001721-199807000-00009>.

26. Delluc A, Gourhant L, Lacut K, et al. Association of common genetic variations and idiopathic venous thromboembolism. Results from EDITh, a hospital-based case-control study. Thromb Haemost. 2010;103(6):1161-9 <http://dx.doi.org/10.1160/TH09-07-0430>.

27. Di Minno MN, Dentali F, Veglia F, Russolillo A, Tremoli E, Ageno W. Antithrombin levels and the risk of a first episode of venous thromboembolism: a case-control study. Thromb Haemost. 2013;109(1):167-9 <http://dx.doi.org/10.1160/TH12-09-0663>.

28. Dimri U, Chatterjee T, Mallhi RS, Philip J, Kushwaha N. Inherited thrombophilia in unprovoked venous thromboembolism: Is non 'O' blood group an additional culprit in Indian patients? Med J Armed Forces India. 2019;75(2):152-7 <http://dx.doi.org/10.1016/j.mjafi.2018.01.008>.

29. Djordjevic V, Rakicevic LJ, Mikovic D, et al. Prevalence of factor V leiden, factor V cambridge, factor II G20210A and methylenetetrahydrofolate reductase C677T mutations in healthy and thrombophilic Serbian populations. Acta Haematol. 2004;112(4):227-9 <http://dx.doi.org/10.1159/000081280>.

30. Farajzadeh M, Bargahi N, Poursadegh Zonouzi A, Farajzadeh D, Pouladi N. Polymorphisms in thrombophilic genes are associated with deep venous thromboembolism in an Iranian population. Meta Gene. 2014;2:505-13 <http://dx.doi.org/10.1016/j.mgene.2014.06.004>.

31. Folsom AR, Cushman M, Tsai MY, Heckbert SR, Aleksic N. Prospective study of the G20210A polymorphism in the prothrombin gene, plasma prothrombin concentration, and incidence of venous thromboembolism. Am J Hematol. 2002;71(4):285-90 <http://dx.doi.org/10.1002/ajh.10229>.

32. Gorski MM, de Haan HG, Mancini I, et al. Next-generation DNA sequencing to identify novel genetic risk factors for cerebral vein thrombosis. Thromb Res. 2018;169:76-81 <http://dx.doi.org/10.1016/j.thromres.2018.06.011>.

33. Heijboer H, Brandjes DP, Buller HR, Sturk A, ten Cate JW. Deficiencies of coagulation-inhibiting and fibrinolytic proteins in outpatients with deep-vein thrombosis. N Engl J Med. 1990;323(22):1512-6 <http://dx.doi.org/10.1056/NEJM199011293232202>.

34. Hillarp A, Zoller B, Svensson PJ, Dahlback B. The 20210 A allele of the prothrombin gene is a common risk factor among Swedish outpatients with verified deep venous thrombosis. Thromb Haemost. 1997;78(3):990-2

35. Jackson A, Brown K, Langdown J, Luddington R, Baglin T. Effect of the angiotensin-converting enzyme gene deletion polymorphism on the risk of venous thromboembolism. Br J Haematol. 2000;111(2):562-4 <http://dx.doi.org/10.1046/j.1365-2141.2000.02408.x>.

36. Jusic-Karic A, Terzic R, Jerkic Z, Avdic A, Podanin M. Frequency and association of 1691 (G>A) FVL, 20210 (G>A) PT and 677 (C>T) MTHFR with deep vein thrombosis in the population of Bosnia and Herzegovina. Balkan J Med Genet. 2016;19(1):43-50 <http://dx.doi.org/10.1515/bjmg-2016-0006>.

37. Karasu A, Engbers MJ, Cushman M, Rosendaal FR, van Hylckama Vlieg A. Genetic risk factors for venous thrombosis in the elderly in a case-control study. J Thromb Haemost. 2016;14(9):1759-64 <http://dx.doi.org/10.1111/jth.13409>.

38. Kalayci D, Gurgey A, Guven D, Parlak H, Hasiripi H. Factor V Leiden and prothrombin 20210 A mutations in patients with central and branch retinal vein occlusion. Acta Ophthalmol Scand. 1999;77(6):622-4 <http://dx.doi.org/10.1034/j.1600-0420.1999.770602.x>.

39. Keijzer MB, den Heijer M, Blom HJ, et al. Interaction between hyperhomocysteinemia, mutated methylenetetrahydrofolatereductase (MTHFR) and inherited thrombophilic factors in recurrent venous thrombosis. Thromb Haemost. 2002;88(5):723-8

40. Kupeli E, Verdi H, Simsek A, Atac FB, Eyuboglu FO. Genetic mutations in Turkish population with pulmonary embolism and deep venous thrombosis. Clin Appl Thromb Hemost. 2011;17(6):E87-94 <http://dx.doi.org/10.1177/1076029610385224>.

41. Legnani C, Palareti G, Guazzaloca G, et al. Venous thromboembolism in young women; role of thrombophilic mutations and oral contraceptive use. Eur Heart J. 2002;23(12):984-90 <http://dx.doi.org/10.1053/euhj.2001.3082>.

42. Lichy C, Dong-Si T, Reuner K, et al. Risk of cerebral venous thrombosis and novel gene polymorphisms of the coagulation and fibrinolytic systems. J Neurol. 2006;253(3):316-20 <http://dx.doi.org/10.1007/s00415-005-0988-4>.

43. Lijfering WM, Middeldorp S, Veeger NJ, et al. Risk of recurrent venous thrombosis in homozygous carriers and double heterozygous carriers of factor V Leiden and prothrombin G20210A. Circulation. 2010;121(15):1706-12 <http://dx.doi.org/10.1161/CIRCULATIONAHA.109.906347>.

44. Lindmarker P, Schulman S, Sten-Linder M, Wiman B, Egberg N, Johnsson H. The risk of recurrent venous thromboembolism in carriers and non-carriers of the G1691A allele in the coagulation factor V gene and the G20210A allele in the prothrombin gene. DURAC Trial Study Group. Duration of Anticoagulation. Thromb Haemost. 1999;81(5):684-9

45. Linna T, Ylikorkala A, Kontula K, Puska P, Tervo T. Prevalence of factor V Leiden in young adults with retinal vein occlusion. Thromb Haemost. 1997;77(1):214-6

46. Mansilha A, Araujo F, Sampaio S, Cunha Ribeiro LM, Braga A. The PORtromb Project: prothrombin G20210A mutation and venous thromboembolism in young people. Cardiovasc Surg. 2002;10(1):45-8 <http://dx.doi.org/10.1016/s0967-2109(00)00150-2>.

47. Mansilha A AF, Severo M, Sampaio SM, Toledo T, Albuquerque R. Combined Factor V Leiden (R506Q) and prothrombin G20210A genotyping in young patients presenting with deep venous thrombosis. Phlebology. 2006;21(1):24-7 <http://dx.doi.org/10.1258/026835506775971171>.

48. Manten B, Westendorp RG, Koster T, Reitsma PH, Rosendaal FR. Risk factor profiles in patients with different clinical manifestations of venous thromboembolism: a focus on the factor V Leiden mutation. Thromb Haemost. 1996;76(4):510-3

49. Marcucci R, Bertini L, Giusti B, et al. Thrombophilic risk factors in patients with central retinal vein occlusion. Thromb Haemost. 2001;86(3):772-6

50. Marcucci R, Bertini L, Liotta AA, et al. Activated protein C resistance is a risk factor for central retinal vein occlusion. Ann Ital Med Int. 2000;15(3):195-8

51. Mitsuguro M, Sakata T, Okamoto A, et al. Usefulness of antithrombin deficiency phenotypes for risk assessment of venous thromboembolism: type I deficiency as a strong risk factor for venous thromboembolism. Int J Hematol. 2010;92(3):468-73 <http://dx.doi.org/10.1007/s12185-010-0687-5>.

52. Nizankowska-Mogilnicka E, Adamek L, Grzanka P, et al. Genetic polymorphisms associated with acute pulmonary embolism and deep venous thrombosis. Eur Respir J. 2003;21(1):25-30 <http://dx.doi.org/10.1183/09031936.03.00034302>.

53. Obeid R, Hakki T, Jouma M, Herrmann W. The risk of venous thromboembolism associated with the factor V Leiden mutation and low B-vitamin status. Clin Chem Lab Med. 2003;41(10):1357-62 <http://dx.doi.org/10.1515/CCLM.2003.208>.

54. Okumus G, Kiyan E, Arseven O, et al. Hereditary thrombophilic risk factors and venous thromboembolism in Istanbul, Turkey: the role in different clinical manifestations of venous thromboembolism. Clin Appl Thromb Hemost. 2008;14(2):168-73 <http://dx.doi.org/10.1177/1076029607305620>.

55. Perez-Ceballos E, Corral J, Alberca I, et al. Prothrombin A19911G and G20210A polymorphisms' role in thrombosis. Br J Haematol. 2002;118(2):610-4 <http://dx.doi.org/10.1046/j.1365-2141.2002.03624.x>.

56. Pestana CI, Torres A, Blanco S, et al. Factor V Leiden and the risk of venous thrombosis, myocardial infarction, and stroke: a case-control study in Venezuela. Genet Test Mol Biomarkers. 2009;13(4):537-42 <http://dx.doi.org/10.1089/gtmb.2008.0100>.

57. Primignani M, Martinelli I, Bucciarelli P, et al. Risk factors for thrombophilia in extrahepatic portal vein obstruction. Hepatology. 2005;41(3):603-8 <http://dx.doi.org/10.1002/hep.20591>.

58. Rahimi Z, Mozafari H, Shahriari-Ahmadi A, et al. Deep venous thrombosis and thrombophilic mutations in western Iran: association with factor V Leiden. Blood Coagul Fibrinolysis. 2010;21(5):385-8 <http://dx.doi.org/10.1097/MBC.0b013e328330e69a>.

59. Renner W, Koppel H, Hoffmann C, et al. Prothrombin G20210A, factor V Leiden, and factor XIII Val34Leu: common mutations of blood coagulation factors and deep vein thrombosis in Austria. Thromb Res. 2000;99(1):35-9 <http://dx.doi.org/10.1016/s0049-3848(00)00219-x>.

60. Ridker PM, Hennekens CH, Miletich JP. G20210A mutation in prothrombin gene and risk of myocardial infarction, stroke, and venous thrombosis in a large cohort of US men. Circulation. 1999;99(8):999-1004 <http://dx.doi.org/10.1161/01.cir.99.8.999>.

61. Rosendaal FR, Koster T, Vandenbroucke JP, Reitsma PH. High risk of thrombosis in patients homozygous for factor V Leiden (activated protein C resistance). Blood. 1995;85(6):1504-8

62. Russo PD, Damante G, Pasca S, Turello M, Barillari G. Thrombophilic mutations as risk factor for retinal vein occlusion: a case-control study. Clin Appl Thromb Hemost. 2015;21(4):373-7 <http://dx.doi.org/10.1177/1076029614522544>.

63. Sakata T, Okamoto A, Mannami T, Matsuo H, Miyata T. Protein C and antithrombin deficiency are important risk factors for deep vein thrombosis in Japanese. J Thromb Haemost. 2004;2(3):528-30 <http://dx.doi.org/10.1111/j.1538-7836.2004.00603.x>.

64. Salazar-Sanchez L, Leon MP, Cartin M, et al. The FXIIIVal34Leu, common and risk factors of venous thrombosis in early middle-age Costa Rican patients. Cell Biochem Funct. 2007;25(6):739-45 <http://dx.doi.org/10.1002/cbf.1389>.

65. Salomon O, Moisseiev J, Rosenberg N, et al. Analysis of genetic polymorphisms related to thrombosis and other risk factors in patients with retinal vein occlusion. Blood Coagul Fibrinolysis. 1998;9(7):617-22 <http://dx.doi.org/10.1097/00001721-199810000-00008>.

66. Shen MC, Lin JS, Tsay W. Protein C and protein S deficiencies are the most important risk factors associated with thrombosis in Chinese venous thrombophilic patients in Taiwan. Thromb Res. 2000;99(5):447-52 <http://dx.doi.org/10.1016/s0049-3848(00)00265-6>.

67. Souto JC, Coll I, Llobet D, et al. The prothrombin 20210A allele is the most prevalent genetic risk factor for venous thromboembolism in the Spanish population. Thromb Haemost. 1998;80(3):366-9

68. Svensson PJ, Zoller B, Mattiasson I, Dahlback B. The factor VR506Q mutation causing APC resistance is highly prevalent amongst unselected outpatients with clinically suspected deep venous thrombosis. J Intern Med. 1997;241(5):379-85 <http://dx.doi.org/10.1046/j.1365-2796.1997.124140000.x>.

69. Tosetto A, Missiaglia E, Frezzato M, Rodeghiero F. The VITA project: prothrombin G20210A mutation and venous thromboembolism in the general population. Thromb Haemost. 1999;82(5):1395-8

70. Tony AM, Vinod V, Nambiar V, Krishnan S, Biswas L. High Prevalence of Plasminogen Activator Inhibitor-1 4G/5G Polymorphism among Patients with Venous Thromboembolism in Kerala, India. Hamostaseologie. 2023;43(2):126-31 <http://dx.doi.org/10.1055/a-1733-2143>.

71. Tregouet DA, Heath S, Saut N, et al. Common susceptibility alleles are unlikely to contribute as strongly as the FV and ABO loci to VTE risk: results from a GWAS approach. Blood. 2009;113(21):5298-303 <http://dx.doi.org/10.1182/blood-2008-11-190389>.

72. Weger M, Renner W, Steinbrugger I, et al. Role of thrombophilic gene polymorphisms in branch retinal vein occlusion. Ophthalmology. 2005;112(11):1910-5 <http://dx.doi.org/10.1016/j.ophtha.2005.05.019>.

73. Weih M, Vetter B, Ziemer S, et al. Increased rate of factor V Leiden mutation in patients with cerebral venous thrombosis. J Neurol. 1998;245(3):149-52 <http://dx.doi.org/10.1007/s004150050195>.

74. Zalavras Ch G, Giotopoulou S, Dokou E, et al. Prevalence of the G20210A prothrombin gene mutation in Northwestern Greece and association with venous thromboembolism. Int Angiol. 2003;22(1):55-7

75. Zerjavic K, Zagradisnik B, Stangler Herodez S, Lokar L, Glaser Krasevac M, Kokalj Vokac N. Is the JAK2 V617F mutation a hallmark for different forms of thrombosis? Acta Haematol. 2010;124(1):49-56 <http://dx.doi.org/10.1159/000314645>.

76. Zhang CL, Li ZM, Song ZH, Song T. Coagulation factor V gene 1691G>A polymorphism as an indicator for risk and prognosis of lower extremity deep venous thrombosis in Chinese Han population. Medicine (Baltimore). 2018;97(22):e10885 <http://dx.doi.org/10.1097/MD.0000000000010885>.

77. Brouwer JL, Veeger NJ, van der Schaaf W, Kluin-Nelemans HC, van der Meer J. Difference in absolute risk of venous and arterial thrombosis between familial protein S deficiency type I and type III. Results from a family cohort study to assess the clinical impact of a laboratory test-based classification. Br J Haematol. 2005;128(5):703-10 <http://dx.doi.org/10.1111/j.1365-2141.2005.05371.x>.

78. Castaman G, Tosetto A, Ruggeri M, Rodeghiero F. Pseudohomozygosity for activated protein C resistance is a risk factor for venous thrombosis. Br J Haematol. 1999;106(1):232-6 <http://dx.doi.org/10.1046/j.1365-2141.1999.01502.x>.

79. Cohen W, Castelli C, Alessi MC, et al. ABO blood group and von Willebrand factor levels partially explained the incomplete penetrance of congenital thrombophilia. Arterioscler Thromb Vasc Biol. 2012;32(8):2021-8 <http://dx.doi.org/10.1161/ATVBAHA.112.248161>.

80. Coppens M, van de Poel MH, Bank I, et al. A prospective cohort study on the absolute incidence of venous thromboembolism and arterial cardiovascular disease in asymptomatic carriers of the prothrombin 20210A mutation. Blood. 2006;108(8):2604-7 <http://dx.doi.org/10.1182/blood-2006-04-016527>.

81. Couturaud F, Kearon C, Leroyer C, et al. Incidence of venous thromboembolism in first-degree relatives of patients with venous thromboembolism who have factor V Leiden. Thromb Haemost. 2006;96(6):744-9

82. De Stefano V, Martinelli I, Mannucci PM, et al. The risk of recurrent deep venous thrombosis among heterozygous carriers of both factor V Leiden and the G20210A prothrombin mutation. N Engl J Med. 1999;341(11):801-6 <http://dx.doi.org/10.1056/NEJM199909093411104>.

83. De Stefano V, Martinelli I, Mannucci PM, et al. The risk of recurrent venous thromboembolism among heterozygous carriers of the G20210A prothrombin gene mutation. Br J Haematol. 2001;113(3):630-5 <http://dx.doi.org/10.1046/j.1365-2141.2001.02827.x>.

84. Di Minno MN, Dentali F, Lupoli R, Ageno W. Mild antithrombin deficiency and risk of recurrent venous thromboembolism: a prospective cohort study. Circulation. 2014;129(4):497-503 <http://dx.doi.org/10.1161/CIRCULATIONAHA.113.003756>.

85. Eichinger S, Weltermann A, Mannhalter C, et al. The risk of recurrent venous thromboembolism in heterozygous carriers of factor V Leiden and a first spontaneous venous thromboembolism. Arch Intern Med. 2002;162(20):2357-60 <http://dx.doi.org/10.1001/archinte.162.20.2357>.

86. Evensen LH, Arnesen CAL, Rosendaal FR, et al. The Risk of Venous Thromboembolism Attributed to Established Prothrombotic Genotypes. Thromb Haemost. 2022;122(7):1221-30 <http://dx.doi.org/10.1055/a-1698-6717>.

87. Hodeib H, Youssef A, Allam AA, et al. Genetic Risk Profiling Associated with Recurrent Unprovoked Venous Thromboembolism. Genes (Basel). 2021;12(6) <http://dx.doi.org/10.3390/genes12060874>.

88. Lijfering WM, Christiansen SC, Rosendaal FR, Cannegieter SC. Contribution of high factor VIII, IX and XI to the risk of recurrent venous thrombosis in factor V Leiden carriers. J Thromb Haemost. 2009;7(11):1944-6 <http://dx.doi.org/10.1111/j.1538-7836.2009.03580.x>.

89. Mahmoodi BK, Brouwer JL, Ten Kate MK, et al. A prospective cohort study on the absolute risks of venous thromboembolism and predictive value of screening asymptomatic relatives of patients with hereditary deficiencies of protein S, protein C or antithrombin. J Thromb Haemost. 2010;8(6):1193-200 <http://dx.doi.org/10.1111/j.1538-7836.2010.03840.x>.

90. Makris M, Leach M, Beauchamp NJ, et al. Genetic analysis, phenotypic diagnosis, and risk of venous thrombosis in families with inherited deficiencies of protein S. Blood. 2000;95(6):1935-41

91. Manderstedt E, Lind-Hallden C, Hallden C, et al. Classic Thrombophilias and Thrombotic Risk Among Middle-Aged and Older Adults: A Population-Based Cohort Study. J Am Heart Assoc. 2022;11(4):e023018 <http://dx.doi.org/10.1161/JAHA.121.023018>.

92. Mateo J, Oliver A, Borrell M, Sala N, Fontcuberta J. Increased risk of venous thrombosis in carriers of natural anticoagulant deficiencies. Results of the family studies of the Spanish Multicenter Study on Thrombophilia (EMET study). Blood Coagul Fibrinolysis. 1998;9(1):71-8 <http://dx.doi.org/10.1097/00001721-199801000-00009>.

93. Mean M, Breakey N, Stalder O, et al. Thrombophilia and outcomes of venous thromboembolism in older patients. Res Pract Thromb Haemost. 2023;7(1):100015 <http://dx.doi.org/10.1016/j.rpth.2022.100015>.

94. Middeldorp S, Meinardi JR, Koopman MM, et al. A prospective study of asymptomatic carriers of the factor V Leiden mutation to determine the incidence of venous thromboembolism. Ann Intern Med. 2001;135(5):322-7 <http://dx.doi.org/10.7326/0003-4819-135-5-200109040-00008>.

95. Miles JS, Miletich JP, Goldhaber SZ, Hennekens CH, Ridker PM. G20210A mutation in the prothrombin gene and the risk of recurrent venous thromboembolism. J Am Coll Cardiol. 2001;37(1):215-8 <http://dx.doi.org/10.1016/s0735-1097(00)01080-9>.

96. Olivo Freites C, Naymagon L. The utility of hereditary thrombophilia testing among patients with unprovoked venous thromboembolism. Int J Lab Hematol. 2022;44(2):393-8 <http://dx.doi.org/10.1111/ijlh.13752>.

97. Pires GS, Ribeiro DD, Oliveira JAQ, et al. Risk factors associated with recurrent venous thromboembolism after a first cerebral venous thrombosis event: A cohort study. Thromb Res. 2019;178:85-90 <http://dx.doi.org/10.1016/j.thromres.2019.04.008>.

98. Puhr HC, Eischer L, Sinkovec H, Traby L, Kyrle PA, Eichinger S. Circumstances of provoked recurrent venous thromboembolism: the Austrian study on recurrent venous thromboembolism. J Thromb Thrombolysis. 2020;49(4):505-10 <http://dx.doi.org/10.1007/s11239-019-01965-z>.

99. Ridker PM, Miletich JP, Stampfer MJ, Goldhaber SZ, Lindpaintner K, Hennekens CH. Factor V Leiden and risks of recurrent idiopathic venous thromboembolism. Circulation. 1995;92(10):2800-2 <http://dx.doi.org/10.1161/01.cir.92.10.2800>.

100. Satpanich P, Rojnuckarin P. Risk factors for venous thromboembolism (VTE) recurrences in Thai patients without cancer. Hematology. 2019;24(1):159-65 <http://dx.doi.org/10.1080/10245332.2018.1535535>.

101. Simioni P, Prandoni P, Lensing AW, et al. The risk of recurrent venous thromboembolism in patients with an Arg506-->Gln mutation in the gene for factor V (factor V Leiden). N Engl J Med. 1997;336(6):399-403 <http://dx.doi.org/10.1056/NEJM199702063360602>.

102. Simioni P, Tormene D, Prandoni P, et al. Incidence of venous thromboembolism in asymptomatic family members who are carriers of factor V Leiden: a prospective cohort study. Blood. 2002;99(6):1938-42 <http://dx.doi.org/10.1182/blood.v99.6.1938>.

103. Tirado I, Mateo J, Soria JM, et al. Contribution of prothrombin 20210A allele and factor V Leiden mutation to thrombosis risk in thrombophilic families with other hemostatic deficiencies. Haematologica. 2001;86(11):1200-8

104. Tormene D, Simioni P, Pagnan A, Prandoni P. The G20210A prothrombin gene mutation: is there room for screening families? J Thromb Haemost. 2004;2(8):1487-8 <http://dx.doi.org/10.1111/j.1538-7836.2004.00858.x>.

105. van Boven HH, Vandenbroucke JP, Briet E, Rosendaal FR. Gene-gene and gene-environment interactions determine risk of thrombosis in families with inherited antithrombin deficiency. Blood. 1999;94(8):2590-4

106. Weingarz L, Schindewolf M, Schwonberg J, et al. Thrombophilia and risk of VTE recurrence according to the age at the time of first VTE manifestation. Vasa. 2015;44(4):313-23 <http://dx.doi.org/10.1024/0301-1526/a000447>.

107. Zoller B, Melander O, Svensson PJ, Engstrom G. Factor V Leiden paradox in a middle-aged Swedish population: A prospective study. Vasc Med. 2018;23(1):52-9 <http://dx.doi.org/10.1177/1358863X17745591>.

108. . !!! INVALID CITATION !!! (7, 9, 14, 18, 25, 28, 33, 34, 44, 51, 53, 55, 58, 67, 80)

109. Alfeel AH, Abdalhabib EK, Mobarki AA, Hamali HA, Ibrahim IK, Elzaki SEG. Frequency of Factor V Leiden and Prothrombin G20210A Mutations in Sudanese Patients with Deep Vein Thrombosis in Khartoum Hospitals. Pakistan Journal of Medical & Health Sciences. 2020;14(3):1091-4

110. Bezgin T, Kaymaz C, Akbal Ö, Yılmaz F, Tokgöz HC, Özdemir N. Thrombophilic Gene Mutations in Relation to Different Manifestations of Venous Thromboembolism: A Single Tertiary Center Study. Clin Appl Thromb Hemost. 2018;24(1):100-6 <http://dx.doi.org/10.1177/1076029616672585>.

111. Daraban AM, Trifa AP, Popp RA, et al. Thrombophilia genetic testing in Romanian young women with acute thrombotic events: role of Factor V Leiden, Prothrombin G20210A, MTHFR C677T and A1298C polymorphisms. Revista Romana De Medicina De Laborator. 2016;24(3):291-305 <http://dx.doi.org/10.1515/rrlm-2016-0032>.

112. Lijfering WM, Middeldorp S, Veeger NJ, et al. Risk of recurrent venous thrombosis in homozygous carriers and double heterozygous carriers of factor V Leiden and prothrombin G20210A. Circulation. 2010;121(15):1706-12 <http://dx.doi.org/10.1161/CIRCULATIONAHA.109.906347>.

113. Mansilha A, Araújo F, Sampaio S, Cunha Ribeiro LM, Braga A. The PORtromb Project: prothrombin G20210A mutation and venous thromboembolism in young people. Cardiovasc Surg. 2002;10(1):45-8 <http://dx.doi.org/10.1016/s0967-2109(00)00150-2>.

114. Mansilha A, Araujo F, Severo M, Sampaio SM, Toledo T, Albuquerque R. Combined Factor V Leiden (R506Q) and prothrombin G20210A genotyping in young patients presenting with deep venous thrombosis. Phlebology. 2006;21(1):24-7 <http://dx.doi.org/10.1258/026835506775971171>.

115. Sakata T, Okamoto A, Mannami T, Matsuo H, Miyata T. Protein C and antithrombin deficiency are important risk factors for deep vein thrombosis in Japanese. Journal of Thrombosis and Haemostasis. 2004;2(3):528-30 <http://dx.doi.org/10.1111/j.1538-7836.2004.00603.x>.

116. Salazar-Sanchez L, Leon MP, Cartin M, et al. The FXIIIVal34Leu, common and risk factors of venous thrombosis in early middle-age Costa Rican patients. Cell Biochemistry and Function. 2007;25(6):739-45 <http://dx.doi.org/10.1002/cbf.1389>.

117. Zhang CL, Li ZM, Song ZH, Song T. Coagulation factor V gene 1691G > A polymorphism as an indicator for risk and prognosis of lower extremity deep venous thrombosis in Chinese Han population. Medicine. 2018;97(22) <http://dx.doi.org/10.1097/md.0000000000010885>.

118. Couturaud F, Kearon C, Leroyer C, et al. Incidence of venous thromboembolism in first-degree relatives of patients with venous thromboembolism who have factor V Leiden. Thromb Haemost. 2006;96(6):744-9

119. Evensen LH, Arnesen CAL, Rosendaal FR, et al. The Risk of Venous Thromboembolism Attributed to Established Prothrombotic Genotypes. Thromb Haemost. 2021 <http://dx.doi.org/10.1055/a-1698-6717>.

120. Hodeib H, Youssef A, Allam AA, et al. Genetic Risk Profiling Associated with Recurrent Unprovoked Venous Thromboembolism. Genes. 2021;12(6) <http://dx.doi.org/10.3390/genes12060874>.

121. Manderstedt E, Lind-Halldén C, Halldén C, et al. Classic Thrombophilias and Thrombotic Risk Among Middle-Aged and Older Adults: A Population-Based Cohort Study. J Am Heart Assoc. 2022;11(4):e023018 <http://dx.doi.org/10.1161/JAHA.121.023018>.

122. Puhr HC, Eischer L, Sinkovec H, Traby L, Kyrle PA, Eichinger S. Circumstances of provoked recurrent venous thromboembolism: the Austrian study on recurrent venous thromboembolism. Journal of Thrombosis and Thrombolysis. 2020;49(4):505-10 <http://dx.doi.org/10.1007/s11239-019-01965-z>.

123. Zöller B, Melander O, Svensson PJ, Engström G. Factor V Leiden paradox in a middle-aged Swedish population: A prospective study. Vasc Med. 2018;23(1):52-9 <http://dx.doi.org/10.1177/1358863X17745591>.
